# Metformin-associated gut microbiota remodeling correlates with reinvigorated splenic immunity in aged mice: microbiome-immune crosstalk via the gut-spleen axis

**DOI:** 10.3389/fimmu.2025.1633486

**Published:** 2025-09-25

**Authors:** Shu-Qin Ding, Xin-Yi Lyu, Shi-Yu Zhou, Yi-Wan Fang, Hao-Xin Ji, Jiang-Yan Li, He-Zuo Lü

**Affiliations:** ^1^ Clinical Laboratory, the First Affiliated Hospital of Bengbu Medical University, Bengbu, Anhui, China; ^2^ The Second Affiliated Hospital, Department of Vascular Surgery, Hengyang Medical School, University of South China, Hengyang, China; ^3^ Anhui Province Key Laboratory of Immunology in Chronic Diseases, Bengbu Medical University, Bengbu, Anhui, China; ^4^ Anhui Province Key Laboratory of Basic and Translational Research of Inflammation-related Diseases, Bengbu Medical University, Bengbu, Anhui, China; ^5^ Anhui Engineering Research Center for Neural Regeneration Technology and Medical New Materials, Bengbu Medical University, Bengbu, Anhui, China

**Keywords:** metformin, gut microbiota, immunosenescence, microbiome-immune crosstalk, gut-spleen axis

## Abstract

**Background and aim:**

Immunosenescence involves age-related immune decline and chronic inflammation, with the spleen serving as a critical hub for immune dysregulation. While gut microbiota influences systemic immunity, its specific role and the potential existence of a gut-spleen axis in mediating splenic aging remains unclear. Therefore, we investigated whether metformin, a microbiota-modulating geroprotective drug, alleviates splenic immunosenescence in aged mice, specifically exploring the link between gut microbiota remodeling and splenic immune rejuvenation.

**Methods:**

Aged C57BL/6 mice (15-month-old) received oral metformin (300 mg/kg/day) or vehicle for 5 months. Systemic toxicity and metabolism were monitored. Splenic immune subsets were analyzed using flow cytometry and immunohistochemistry. Gut microbiota composition (16S rRNA sequencing), cytokine levels (RT-qPCR), and functional pathways were assessed.

**Results:**

Metformin caused no hepatorenal toxicity or weight changes. Treated mice exhibited increased cytotoxic T cells (Tc) and macrophages in the spleen, with reduced Th/Tc ratios and M1/M2 polarization. Pro-inflammatory cytokines (Ifng, Il17a, Il1b, Il6) decreased, while anti-inflammatory markers (Arg1, Tgfb1) rose. Gut microbiota showed enriched *Akkermansia*, *Muribaculum*, and *Duncaniella*, but reduced *Lactobacillus*. *Akkermansia/Muribaculum* negatively correlated with pro-inflammatory cytokines, whereas *Lactobacillus* and *Lachnospiraceae* linked to pro-inflammatory responses. Functional prediction analysis based on 16S rRNA sequencing data indicated upregulation of bile acid metabolism and oxidative phosphorylation pathways.

**Conclusion:**

Metformin reshapes the gut microbiota, which is associated with mitigation of age-associated splenic immune dysregulation, favoring anti-inflammatory macrophage polarization and cytotoxic T cell expansion. Critically, our findings establish the gut-spleen axis as a key mediator of splenic immunosenescence and a novel therapeutic target, which positions metformin as a promising microbiota-directed geroprotective agent. Future research should prioritize mechanistic dissection of gut-spleen communication and clinical validation of metformin’s geroprotective efficacy in human populations.

## Introduction

The progressive decline in immune function with advancing age, termed immunosenescence, is a hallmark of aging that significantly increases susceptibility to infections, diminishes vaccine efficacy, and elevates risks of chronic inflammatory diseases and malignancies (1–4). This phenomenon is characterized by a complex interplay of cellular and molecular alterations across both innate and adaptive immune systems. Among lymphoid organs, the spleen serves as a critical hub for systemic immune surveillance, orchestrating responses to blood-borne pathogens and maintaining immune homeostasis (5). In aged individuals, the spleen undergoes profound structural and functional remodeling, marked by atrophy of white pulp compartments (e.g., periarteriolar lymphoid sheaths and germinal centers), skewed lymphocyte subset ratios, and accumulation of senescent immune cells (6). These changes collectively contribute to impaired antigen presentation, reduced lymphocyte proliferation, and dysregulated cytokine production, culminating in compromised host defense and heightened systemic inflammation (inflammaging) (7, 8). While extensive research has focused on intrinsic immune cell aging mechanisms, emerging evidence highlights the gut microbiota as a pivotal extrinsic modulator of systemic immunity, particularly in the context of aging (9).

The gut microbiota, a dynamic ecosystem of trillions of microorganisms, engages in bidirectional crosstalk with the host immune system through metabolite production, pathogen-associated molecular pattern (PAMP) signaling, and direct microbial-host cell interactions (10, 11). Age-related dysbiosis, characterized by reduced microbial diversity, depletion of beneficial taxa (e.g., *Bifidobacterium, Lactobacillus*), and expansion of pathobionts (e.g., *Enterobacteriaceae*), has been implicated in exacerbating immunosenescence (12). Mechanistically, gut-derived microbial metabolites such as short-chain fatty acids (SCFAs), secondary bile acids (SBAs), and tryptophan derivatives exert systemic immunomodulatory effects by influencing hematopoietic stem cell differentiation, T-cell polarization, and macrophage function (13, 14). Conversely, translocation of pro-inflammatory bacterial components (e.g., lipopolysaccharides) through a “leaky” aged intestinal barrier may fuel chronic low-grade inflammation (15). Notably, the spleen, despite lacking direct anatomical continuity with the gut, receives substantial microbial signals via circulating metabolites and immune cells primed in gut-associated lymphoid tissues (GALT) (16). This gut-spleen axis positions the microbiota as a potential therapeutic target to rejuvenate aged splenic immunity.

Metformin, a first-line oral antidiabetic drug, has garnered increasing attention for its pleiotropic anti-aging and immunomodulatory properties beyond glycemic control (17, 18). Preclinical studies demonstrate that metformin extends health span in model organisms, attenuates age-related chronic inflammation, and enhances vaccine responses in elderly populations (19–21). While its direct effects on immune cells, such as AMP-activated protein kinase (AMPK)-mediated suppression of NLRP3 inflammasome activation and promotion of autophagy, are well-documented (22, 23), recent evidence suggests that metformin’s systemic benefits may be partially mediated through gut microbiota modulation (24). Metformin treatment consistently enriches SCFA-producing bacteria and reduces proteobacterial loads in diabetic and aged models (24, 25). These microbial shifts correlate with improved intestinal barrier integrity and attenuated systemic inflammation (24, 26).

However, critical knowledge gaps persist regarding whether metformin-induced microbiota remodeling can functionally restore immune microenvironments in aged lymphoid organs, particularly the spleen—a question with profound implications for developing microbiota-targeted therapies against immunosenescence. Given that age-related microbiota alterations differ qualitatively from those in metabolic disorders, it remains unclear whether metformin exerts consistent or divergent microbial modulatory effects in geriatric populations. Addressing these questions is essential to evaluate metformin’s translational potential as a geroprotective agent targeting both metabolic and immune aging. This study aims to delineate the tripartite relationship between metformin, gut microbiota, and splenic immune microenvironment in aged mice.

## Methods

### Animals and experimental design

Using the online RNASeqPower Sample Size Calculator (https://rodrigo-arcoverde.shinyapps.io/rnaseq_power_calc/), a sample size of n = 11 was determined to achieve 89.03% statistical power (α = 0.05). This sample size ensures both statistical validity for 16S rRNA sequencing and compliance with animal ethics requirements. Therefore, a total of 22 specific pathogen-free (SPF) healthy male C57BL/6 mice (15-month-old, weighing 30.46 ± 3.05g) were obtained from Chang Zhou Cavens Laboratory Animal Ltd. The mice were housed under standardized conditions (12-hour light/dark cycle, 22 ± 1°C, 50-60% humidity) with ad libitum access to water and a standard chow diet. Mice were randomly divided into two groups: (1) Control group (CON, n = 11): administered vehicle (sterile water) via oral gavage daily; (2) Metformin-treated group (TEST, n = 11): administered metformin (Sangon biotech, Shanghai, China) dissolved in sterile water at 300 mg/kg body weight/day via oral gavage for 5 months. Body weight was monitored weekly. All experimental procedures were approved by the Animal Care Ethics Committee of Bengbu Medical University. The Animal Ethical Approval number was 2020-050.

### Sample collection and processing

At 20 months of age, fecal samples were collected and stored at -80°C for microbiota analysis. After specimen collection, the mice were fasted for 6 hours and euthanized by CO_2_ asphyxiation. Euthanasia was induced using a small animal gas anesthesia system (Yuyan Scientific Instrument Co., Ltd, Shanghai, China). Animals were placed in a sealed chamber, and compressed CO_2_ was introduced at a flow rate of 30% of the chamber volume per minute. Once deep anesthesia was confirmed by the absence of a pedal reflex (toe pinch) and respiratory arrest, mice were promptly removed from the chamber. Terminal blood collection was performed via cardiac puncture while the animals remained under deep anesthesia. Death was confirmed following blood collection by cervical dislocation or exsanguination. Blood was centrifuged (3,000 × g, 15min, 4°C) for isolate serum, and stored at -80°C for biochemical analysis. Spleens were aseptically excised, weighed, and divided into three portions for distinct processing protocols. For RNA real-time quantitative polymerase chain reaction (RT-qPCR) analysis, tissues were either processed immediately or flash-frozen in liquid nitrogen followed by storage at -80°C. For flow cytometric analysis, spleen tissues were mechanically dissociated by pressing through a 45-μm nylon mesh using a syringe plunger. The resulting cell suspensions underwent purification through Percoll gradient centrifugation (Solarbio, Beijing, China). For histological processing, spleens were post-fixed in 4% PFA for 12 hours at 4°C, then cryoprotected by immersion in 30% sucrose solution (prepared in 0.01 M PBS, pH 7.4) for 24 hours at 4°C. Tissues were embedded in optimal cutting temperature (OCT) compound (Tissue-Tek, Miles, Elkart, IN) and sectioned into 6-μm slices using a cryostat (Leica CM1900, Bannockburn, IL). Sections were mounted on poly-L-lysine-coated slides and stored at -80°C until further processing for staining.

### Serum biochemical analysis

Hepatic, renal, and metabolic parameters were quantified using commercial assay kits: alanine aminotransferase (ALT), aspartate aminotransferase (AST), total bilirubin (TBIL), cholinesterase (CHE), creatinine (CRE), urea (UREA), albumin (ALB), globulin (GLB), creatine kinase (CK), and lactate dehydrogenase (LDH) (all from Ortho-clinical diagnostics, inc., NY, USA). Measurements were performed on a VITROS 5600 Integrated System (Ortho-clinical diagnostics, inc.) following manufacturer protocols.

### 16S rRNA gene sequencing and microbiota analysis

Fecal DNA was extracted using the QIAamp DNA Stool Mini Kit (QIAGEN, Hilden, Germany). The 16S rRNA gene was amplified with primers 27F (5’-AGRGTTYGATYMTGGCTCAG-3’) and 1492R (5’-RGYTACCTTGTTACGACTT-3’) and sequenced on an Illumina NovaSeq 6000 platform (2 × 250 bp). Raw reads were processed in QIIME2 (v2021.11) using DADA2 for denoising and amplicon sequence variant (ASV) clustering. Taxonomic assignment was performed against the SILVA (v138) database.

Bioinformatic analysis of the gut microbiota was carried out using the Majorbio Cloud platform (https://cloud.majorbio.com).

Rarefaction curves were calculated with Mothur v1.30.1. Alpha diversities (Chao1 and Shannon) were analyzed with R-3.3.1 (stat) package. Hierarchical clustering and principal coordinate analysis (PCoA) were analyzed with R-3.3.1 (vegan) based on Bray-curtis dissimilarity. ANOSIM analysis was used to confirm statistically significant separation between groups. Beta diversity difference analysis performed with python-2.7 package and Wilcoxon rank-sum test. The linear discriminant analysis (LDA) effect size (LEfSe) (http://huttenhower.sph.harvard.edu/LEfSe) was performed to identify the significantly abundant taxa of bacteria between the two groups (LDA score > 2, *p*<0.05).

Putative functional profiles for Clusters of Orthologous Groups (COG) categories and Kyoto Encyclopedia of Genes and Genomes (KEGG) pathways were inferred from 16S rRNA gene amplicon sequences using PICRUSt2 (Phylogenetic Investigation of Communities by Reconstruction of Unobserved States, version 2.5.2). The PICRUSt2 workflow involved placing ASVs into a reference phylogenetic tree, followed by hidden-state prediction of gene family copy numbers for KEGG orthologs (KOs) and COG categories. The predicted copy numbers were then multiplied by ASV abundance counts to generate metagenome predictions. For KEGG pathways, differential abundance testing for pathways was performed via ANCOM-BC2 (QIIME2 plugin). Differentially abundant KEGG pathways (Pathway Level 3) were filtered using a Benjamini-Hochberg-adjusted p-value threshold of ≤ 0.05. Only pathways meeting this criterion were included. All significantly enriched pathways were systematically curated by cross-referencing them with established prokaryotic metabolic capabilities using MetaCyc’s bacterial pathway database (https://metacyc.org/) and literature evidence. Pathways with only eukaryotic associations were excluded from biological interpretation. COG category abundances were normalized as relative abundances per sample.

Correlation analyses were performed using R-3.3.1, python-2.7 package. In the correlation analysis between gut microbiota composition and clinical factors, dominant bacterial genera were operationally defined as the top 10 most abundant genera at the genus level based on mean relative abundance across all samples. This selection criterion ensured inclusion of taxa with the highest biological relevance to community structure. Correlations were assessed using Spearman’s rank correlation coefficient (ρ), a non-parametric method chosen for its robustness against non-normally distributed data and ability to capture monotonic relationships. Statistical significance (p-values) was derived from the Spearman test statistic, with the null hypothesis of no correlation rejected at *p*<0.05. To mitigate false discovery risks inherent in multiple hypothesis testing, all *p*-values underwent rigorous adjustment via the Benjamini-Hochberg false discovery rate (FDR) correction procedure. This approach controlled the expected proportion of false positives among significant results at α = 0.05. The final significance threshold for correlations was FDR-adjusted *p*<0.05, with correlation strength and direction visualized in heatmaps.

### Flow cytometry

Single-cell suspensions were stained with fluorochrome-conjugated antibodies and corresponding isotype-matched controls (detailed in **Table 1**). Briefly, antibody cocktails containing isotype controls and specific antibodies, at manufacturer-recommended concentrations, were added to 200 µL aliquots of cell suspension. Following 30-minute incubation at room temperature with light protection, cells underwent two successive washes with 5 mL phosphate-buffered saline (PBS) using centrifugation (400 ×g, 5min). Washed cells were fixed in 2% paraformaldehyde (PFA) in PBS (pH 7.4) for subsequent analysis. Flow cytometry acquisition was performed using a flow cytometer (RaiseCare Biotechnology Co., Ltd., Qingdao, China) with standardized voltage settings. Data analysis was conducted using Raiseflower software (v2.1.3, RaiseCare Biotechnology). For the flow cytometry gating strategy, gates defining positive populations were established based on fluorescence thresholds set using isotype-matched control antibodies (**Supplementary Figure S1**). The hierarchical gating approach first selected singlet events via FSC-A/FSC-H. Subpopulations were subsequently gated using lineage-defining markers: CD3^+^ for T cells, with CD4^+^ and CD8^+^ subsets (**Supplementary Figure S1A**); CD3^-^B220^+^ for B cells and CD3^-^NK1.1^+^ for NK cells (**Supplementary Figure S1B**); and CD45^+^Ly-6G^+^ for neutrophils and CD45^+^F4/80^+^ for macrophages (**Supplementary Figure S1C**). Macrophage subsets were further classified as CD68^+^CD86^+^ (M1) and CD68^+^CD163^+^ (M2) based on established polarization markers (**Supplementary Figure S1D**).

**Table 1:** Antibodies used in this study.

Antigen	Host Species and Clone	Cat. # or Lot#	RRID	Conjugation	Source	Used concentration	Methods
CD3	rat monoclonal	12-0032-82	AB_2811741	PE	Invitrogen	0.25 μg/test	FCM
F4/80	rat monoclonal	11-4801-82	AB_2637191	FITC		0.5 μg/test	
CD4	rat monoclonal	11-0041-82	AB_464892	FITC		0.25 μg/test	
CD45	rat monoclonal	A15395	AB_2534409	APC-Cyanine7		0.125 μg/test	
B220	rat monoclonal	17-0452-82	AB_469395	APC		0.125 μg/test	
Ly-6G	rat monoclonal	17-9668-82	AB_2573307	APC		0.125 μg/test	
CD68	rat monoclonal	MA5-16676	AB_2538170	FITC		0.25 μg/test	
CD86	rat monoclonal	17-0862-82	AB_469419	APC		0.125 μg/test	
CD163	rat monoclonal	12-1631-82	AB_2716924	PE		0.25 μg/test	
IgG2a kappa Isotype Control	rat	17-4321-81	AB_470181	APC		0.125 μg/test	
IgG2a kappa	rat	47-4321-82	AB_1271997	APC-eFluor™ 780		0.25 μg/test	
IgG2b kappa Isotype Control	rat	12-4031-82	AB_470042	PE		0.25 μg/test	
IgG2b kappa Isotype Control	rat	11-4321-80	AB_1834375	FITC		0.25 μg/test	
CD3	rat monoclonal	14-0032-82	AB_467053		Invitrogen	1:200	IHF
CD19	mouse monoclonal	14-0199-82	AB_467151				
Ly-6G	rabbit polyclonal	PA5-141170	AB_2932621				
F4/80	rabbit monoclonal	MA5-16363	AB_2537882				
CD68	rat Monoclonal	14-0681-82	AB_2572857				
Arg1	rabbit polyclonal	PA5-29645	AB_2547120				
CD86	rabbit polyclonal	PA5-79007	AB_2746123				
NK1.1	mouse monoclonal	MA1-70100	AB_2296673				
CD45	rat monoclonal	14-0451-82	AB_467251				
Rat IgG (H+L)	goat polyclonal	31629	AB_228240	FITC			
Rat IgG (H+L)	goat polyclonal	A-21434	AB_2535855	Alexa Fluor™ 555			
Mouse IgG (H+L)	goat polyclonal	62-6511	AB_2533946	FITC			
Mouse IgG (H+L)	goat polyclonal	A-11032	AB_2534091	Alexa Fluor™ 594			
Rabbit IgG (H+L)	goat polyclonal	A-11008	AB_143165	Alexa Fluor™ 488			
Rabbit IgG (H+L)	donkey polyclonal	A-21207	AB_141637	Alexa Fluor™ 594			

### Immunohistochemical fluorescence

The immunofluorescence (IHF) assay was conducted following established protocols (21, 22). Briefly, frozen spleen sections fixed in 4% paraformaldehyde (PFA/PBS) were incubated with primary antibodies (see **Table 1** for specifications) at 4 °C for 16–18 hours in a humidified chamber. Following three 5-minute washes with 0.01 M PBS (pH 7.4), sections were incubated with corresponding fluorescein-conjugated secondary antibodies (refer to **Table 1** for dilutions) for 1 hour at room temperature under light-protected conditions. After subsequent PBS washes, slides were mounted using ProLong™ Gold Antifade Mountant containing DAPI nuclear counterstain (Thermo Fisher Scientific, Waltham, MA, USA) and sealed with nail polish. Fluorescence imaging was performed using an Axio Observer Z1 inverted microscope equipped with ApoTome.2 structured illumination (Carl Zeiss AG, Oberkochen, Germany). All images were acquired with consistent exposure settings using ZEN Blue 3.1 software (Zeiss).

### RNA extraction and RT-qPCR

Total RNA was isolated from murine spleen tissues using TRIzol™ Reagent (Thermo Fisher Scientific, Carlsbad, CA, USA) following the manufacturer’s protocol. RNA integrity was verified using an Agilent 2100 Bioanalyzer with RNA Integrity Numbers (RIN) > 7.0, and quantification was performed using a NanoDrop 2000 spectrophotometer (Thermo Fisher Scientific) with A260/A280 ratios between 1.8 and 2.0. First-strand cDNA synthesis was conducted using the BeyoRT™ II First Strand cDNA Synthesis Kit with gDNA Eraser (Beyotime Biotechnology, Shanghai, China), starting with 1 μg total RNA input. Quantitative PCR amplification was performed in triplicate reactions using BeyoFast™ SYBR Green qPCR Mix (Beyotime Biotechnology) on an Applied Biosystems 7500 Real-Time PCR System (Thermo Fisher Scientific). Primer sequences are detailed in **Table 2**. We specifically selected qPCR targets to profile polarized immune pathways relevant to microbiome-immune crosstalk. These targets represent key T helper subsets: Th1 (*Ifng*), Th2 (*Il4* and *Il10*), Th17 (*Il17a*), and Treg (*Foxp3*); and macrophage polarization states: M1 (*Il1b* and *Il6*) and M2 (*Arg1* and *Tgfb1*). This selection of canonical cytokine genes reflects functional axes known to be altered in gut-microbiota interactions (27, 28). Although markers such as *Il21*, *Cxcl13*, *Il2*, or NF-κB pathway components could provide supplementary insights, our focused panel aligns directly with the study’s core hypothesis centered on T cell and macrophage polarization. Gene expression quantification was calculated using the comparative 2−^ΔΔCt^ method with normalization to the Gapdh reference gene (29).

**Table 2:** Primers used in this study.

Gene	Forward primer 5′ - 3′	Reverse primer 5′ - 3′
*Il1b*	CACTACAGGCTCCGAGATGAACAAC	TGTCGTTGCTTGGTTCTCCTTGTAC
*Il6*	CTCCCAACAGACCTGTCTATAC	CCATTGCACAACTCTTTTCTCA
*Ifng*	CTTGAAAGACAATCAGGCCATC	CTTGGCAATACTCATGAATGCA
*Il17a*	GAGCTTCATCTGTGTCTCTGAT	GCCAAGGGAGTTAAAGACTTTG
*Il4*	TACCAGGAGCCATATCCACGGATG	TGTGGTGTTCTTCGTTGCTGTGAG
*Il10*	TTCTTTCAAACAAAGGACCAGC	GCAACCCAAGTAACCCTTAAAG
*Foxp3*	GGCAGAGAGGTATTGAGGGTG	CTTTCTTCTGTCTGGAGTGGC
*Tgfb1*	ACTGGAGTTGTACGGCAGTG	GGGGCTGATCCCGTTGATTT
*Arg1*	CATATCTGCCAAAGACATCGTG	GACATCAAAGCTCAGGTGAATC
*Gapdh*	AATGTGTCCGTCGTGGATCTGA	AGTGTAGCCCAAGATGCCCTTC

### Statistical analysis

Data are presented as mean ± SD. Group comparisons were performed using unpaired Student’s t-test or Wilcoxon rank-sum test for parametric and non-parametric data, respectively. Multiple testing corrections were applied via the Benjamini-Hochberg method. Correlations between microbiota and immune parameters were assessed using Spearman’s rank correlation. Statistical significance was set at *p*<0.05. All analyses were performed in GraphPad Prism v9.3.1 or R v4.1.2.

## Results

### Effects of long-term metformin treatment on body weight and serum biochemical parameters in aged mice

As summarized in **Table 3**, the baseline body weight of 15-month-old mice did not differ significantly between the control (30.43 ± 2.85g) and metformin-treated (30.50 ± 3.37g) groups (unpaired t-test, *p*=0.96, n = 11). After 5 months of oral metformin administration (300 mg/kg/day), body weights remained comparable between groups (34.15 ± 3.38g vs. 32.70 ± 4.10g; *p*=0.38, n = 11), indicating no treatment-related effects on growth or systemic metabolism.

**Table 3:** Effects of long-term metformin treatment on body weight and serum biochemical parameters in aged mice.

Parameter	CON (n = 11)	TEST (n = 11)	p value
Body weight (g) (before treatment)	30.43 ± 2.85	30.50 ± 3.37	0.96
Body weight (g) (after treatment)	34.15 ± 3.38	32.70 ± 4.10	0.38
ALT (U/L)	51.18 ± 30.13	51.82 ± 21.10	0.95
AST (U/L)	132.09 ± 95.85	133.45 ± 75.99	0.97
AST/ALT	3.82 ± 3.49	3.07 ± 2.06	0.52
CHE (U/L)	3111.82 ± 1187.19	2403.76 ± 1098.53	0.15
TBIL (μmol/L)	3.44 ± 1.24	3.24 ± 1.51	0.71
ALB (g/L)	26.62 ± 9.13	23.87 ± 4.40	0.41
GLB (g/L)	26.57 ± 4.04	25.93 ± 4.05	0.70
A/G	1.04 ± 0.46	0.94 ± 0.23	0.54
CRE (μmol/L)	23.45 ± 6.64	28.18 ± 8.77	0.18
UREA (mmol/L)	12.96 ± 1.88	12.22 ± 1.20	0.29
CK (U/L)	1343.00 ± 999.57	1437.36 ± 1126.96	0.83
LDH (U/L)	839.55 ± 231.24	913.82 ± 336.69	0.54

ALT, alanine aminotransferase; AST, aspartate aminotransferase; CHE, cholinesterase; TBIL, total bilirubin; ALB, albumin; GLB, globulin; A/G, albumin-to-globulin ratio; CRE, creatinine; UREA, blood urea nitrogen; CK, creatine kinase; LDH, lactate dehydrogenase. Data presented as mean ± SD. Statistical analyses were conducted using the unpaired t-test for normally distributed data (body weight) and the Mann-Whitney U test for non-normally distributed biochemical parameters. No significant differences were observed between groups for any parameter (*p* > 0.05).

Serum analyses revealed no treatment-related toxicity across hepatic, renal, or cardiac systems. Hepatic function markers, including alanine aminotransferase (ALT: 51.18 ± 30.13 U/L vs. 51.82 ± 21.10 U/L; *p*=0.95, n = 11) and aspartate aminotransferase (AST: 132.09 ± 95.85 U/L vs. 133.45 ± 75.99 U/L; *p*=0.97, n = 11), showed no significant differences. The AST/ALT ratio (3.82 ± 3.49 vs. 3.07 ± 2.06; *p*=0.52, n = 11), total bilirubin (TBIL: 3.44 ± 1.24 μmol/L vs. 3.24 ± 1.51 μmol/L; *p*=0.71, n = 11), and cholinesterase (CHE: 3111.82 ± 1187.19 U/L vs. 2403.76 ± 1098.53 U/L; *p*=0.15, n = 11) remained unaffected. Renal function parameters, creatinine (CRE: 23.45 ± 6.64 μmol/L vs. 28.18 ± 8.77 μmol/L; *p*=0.18, n = 11) and urea (UREA: 12.96 ± 1.88 mmol/L vs. 12.22 ± 1.20 mmol/L; *p*=0.29, n = 11), also exhibited no statistically significant changes. Protein metabolism markers, albumin (ALB: 26.62 ± 9.13 g/L vs. 23.87 ± 4.40 g/L; *p*=0.41, n = 11), globulin (GLB: 26.57 ± 4.04 g/L vs. 25.93 ± 4.05 g/L; *p*=0.70, n = 11), and the albumin/globulin ratio (A/G: 1.04 ± 0.46 vs. 0.94 ± 0.23; *p*=0.54, n = 11), were similarly unaltered. Cardiac and muscle injury markers, creatine kinase (CK: 1343.00 ± 999.57 U/L vs. 1437.36 ± 1126.96 U/L; *p*=0.83, n = 11) and lactate dehydrogenase (LDH: 839.55 ± 231.24 U/L vs. 913.82 ± 336.69 U/L; *p*=0.54, n = 11), showed no treatment-associated elevations. Collectively, these data demonstrate that prolonged metformin treatment at the tested dosage does not induce systemic toxicity or clinically significant perturbations in metabolic or organ function in aged mice.

### The diversities of the gut microbiota

**Figure 1:**
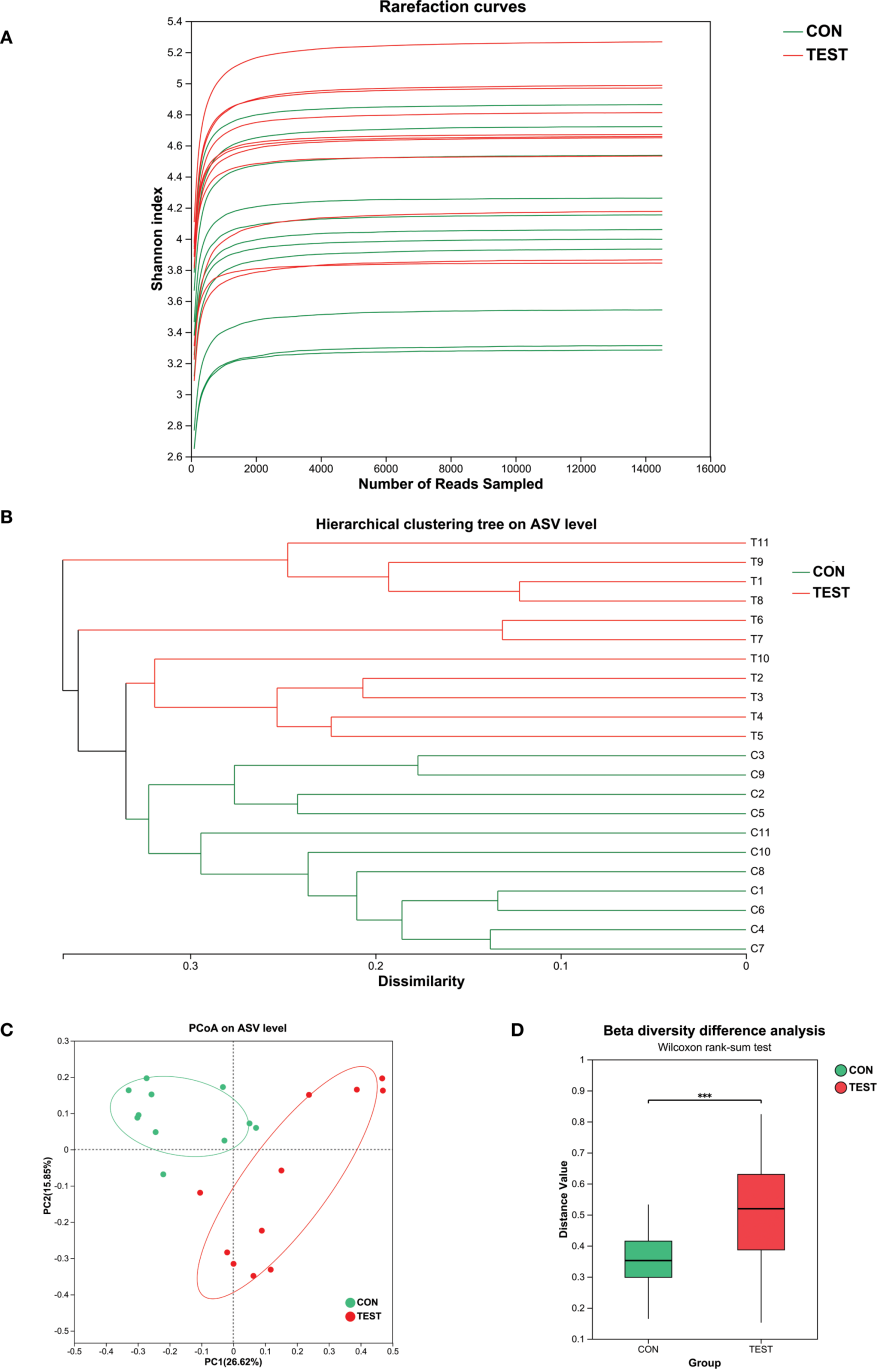
The diversities of the gut microbiota. **(A)** Rarefaction curve; **(B)** Hierarchical clustering analysis. Metformin-treated samples (TEST, green) formed a distinct clade separate from controls (CON, red); **(C)** PCoA analysis. CON, control group; TEST, Metformin-treated group; **(D)** Beta diversity difference analysis (R=0.48, ***p<0.001, Wilcoxon rank-sum test).

To delineate the tripartite relationship between long-term metformin treatment, gut microbiota, and splenic immune microenvironment in aged mice, 16S rRNA gene sequencing was employed to investigate metformin-induced alterations in gut microbiota composition. **Supplementary Tables S1** and **S2** showed the post-processed 16S-count at ASV- and genus-level, respectively. The rarefaction curves in **Figure 1A** reached plateaus as sequencing depth increased, indicating adequate coverage for capturing species diversity. This asymptotic pattern demonstrated sufficient sequencing depth for downstream analyses. Hierarchical clustering analysis using Bray-Curtis dissimilarity revealed distinct microbial composition-based segregation between two groups (**Figure 1B**). To assess the taxonomic richness and evenness of the gut microbiota community, we performed alpha diversity analysis. The Chao1 and Shannon indices were used to evaluate sequencing depth adequacy and quantify species diversity, respectively. The Chao1 index showed no significant difference between the two groups (**Supplementary Figure S2A**, *p* > 0.05). In contrast, the Shannon index revealed significantly higher diversity in the metformin-treated group (**Supplementary Figure S2B**, *p*<0.05). This indicates that while sequencing depth was comparable between groups, the metformin-treated group exhibited greater species diversity. Subsequently, beta diversity analysis was used to measure differences in community composition between the two groups. The dendrogram topology showed tighter clustering of biological replicates, reflecting high intra-group similarity. Color-coding according to experimental conditions confirmed metformin-treated samples (TEST, green) formed a distinct clade separate from controls (CON, red). Principal coordinates analysis (PCoA) based on Bray-Curtis dissimilarity revealed significant β-diversity patterns between control and treatment groups (**Figures 1C, D**). The two-dimensional ordination plot illustrated distinct clustering patterns, where sample proximity reflected similarity in microbial community composition (**Figure 1C**). ANOSIM analysis confirmed statistically significant separation between groups (**Figure 1D**, R=0.48, *p*=0.001), with inter-group distances substantially exceeding intra-group variations.

### Modulation of the gut microbiota by metformin

**Figure 2:**
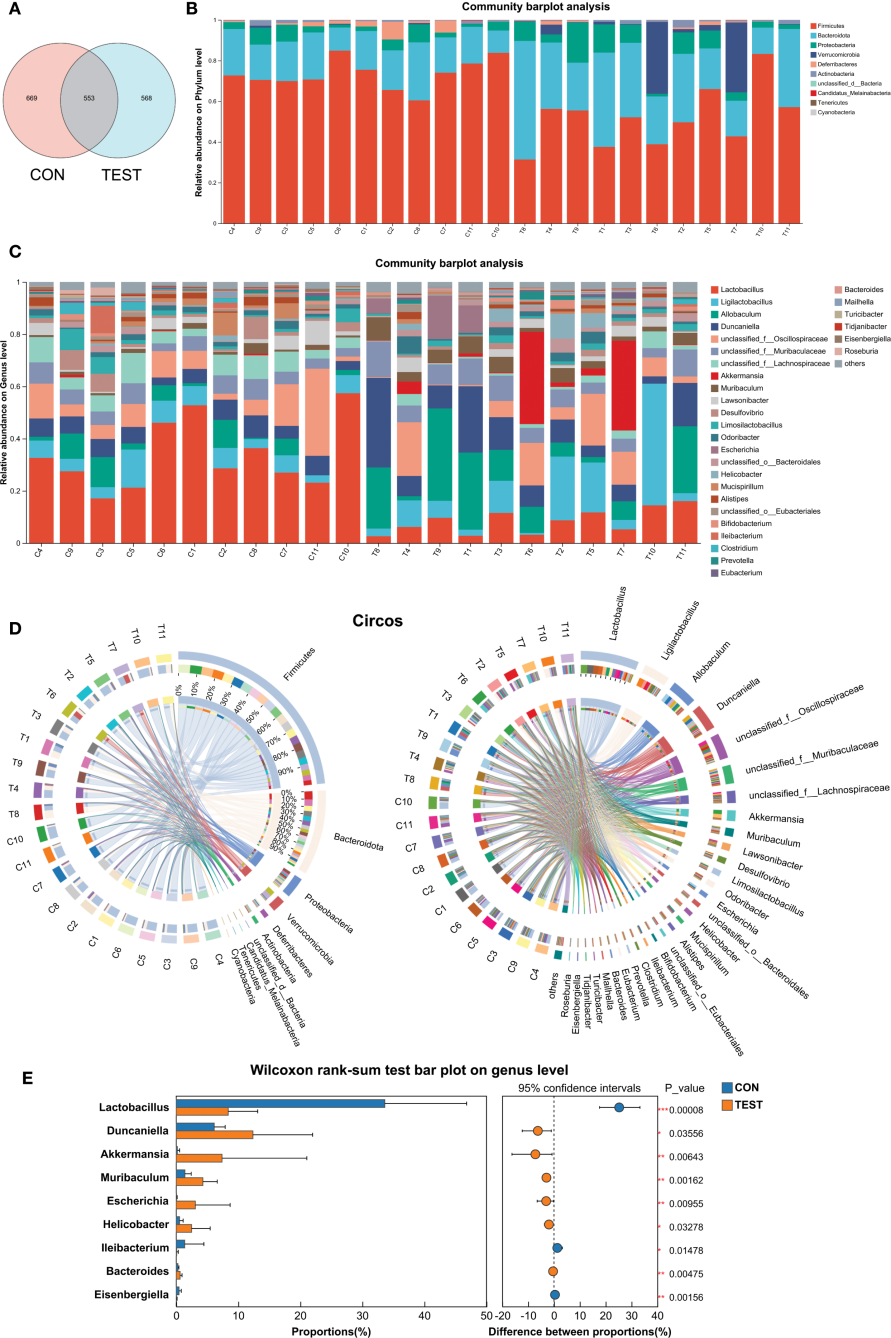
Modulation of the gut microbiota by metformin. **(A)** Venn diagram of ASV; (B and C) Bar diagram at phylum level **(B)** and genus level **(C)**; **(D)** Circos diagram at phylum level (left) and genus level (right). **(E)** Differentially abundant genera between CON and TEST groups were compared by Wilcoxon rank-sum test (*p<0.05, **p<0.01, ***p<0.001). CON, control group; TEST, Metformin-treated group.

Metformin-induced gut microbiota modulation was characterized through amplicon sequence variant (ASV) distribution analysis and taxonomic profiling. Venn diagram analysis revealed group-specific ASV patterns, with 1,790 ASVs identified across cohorts, comprising 669 control-exclusive (CON) and 568 metformin-exclusive (TEST) variants, while 553 ASVs (30.9% of total) were shared (**Figure 2A**). QIIME2-processed data (DADA2 denoising, 99% identity clustering) demonstrated phylum-level restructuring, where CON microbiota was dominated by *Firmicutes* (73.55 ± 7.37%), *Bacteroidetes* (18.73 ± 5.18%), and *Proteobacteria* (4.18 ± 2.93%), collectively representing 96.45% of community composition. In Metformin treatment group (TEST), the above three microbial communities still dominate, with proportions of 51.82 ± 14.48%, 31.18 ± 13.44%, and 7.82 ± 5.78%, respectively. Metformin treatment also significantly increased *Verrucomicrobia* abundance from 0.26% to 7.31%, establishing a four-phylum dominance pattern (**Figures 2B, D**).

Genus-level analysis (threshold: relative abundance >2%, prevalence >80% samples) identified differential taxa through ANCOM-BC2. Metformin-treated mice exhibited increased proportions of *Muribaculum* (Fold Change, FC=3.08), *Akkermansia* (FC=37.58), *Escherichia* (FC=50.70), *Helicobacter* (FC=4.58), *Duncaniella* (FC=2.02), and *Allobaculum* (FC=2.95). Conversely, *Lactobacillus* (FC=0.25), *unclassified Lachnospiraceae* (FC=0.33), *Desulfovibrio* (FC=0.33), and *Mucispirillum* (FC=0.27) were significantly decreased (*p*<0.05, **Figures 2C, D**). The above results were further validated by Wilcoxon rank-sum test (*p*<0.05, **Figure 2E**). For microbiome visualization, the ANCOM-BC2 results with a heatmap showing relative abundances of differentially abundant genera were shown in **Supplementary Figure S3**.

These findings suggest that metformin can modulate the intestinal microbiota composition in mice.

### Species differences and marker species analysis

**Figure 3:**
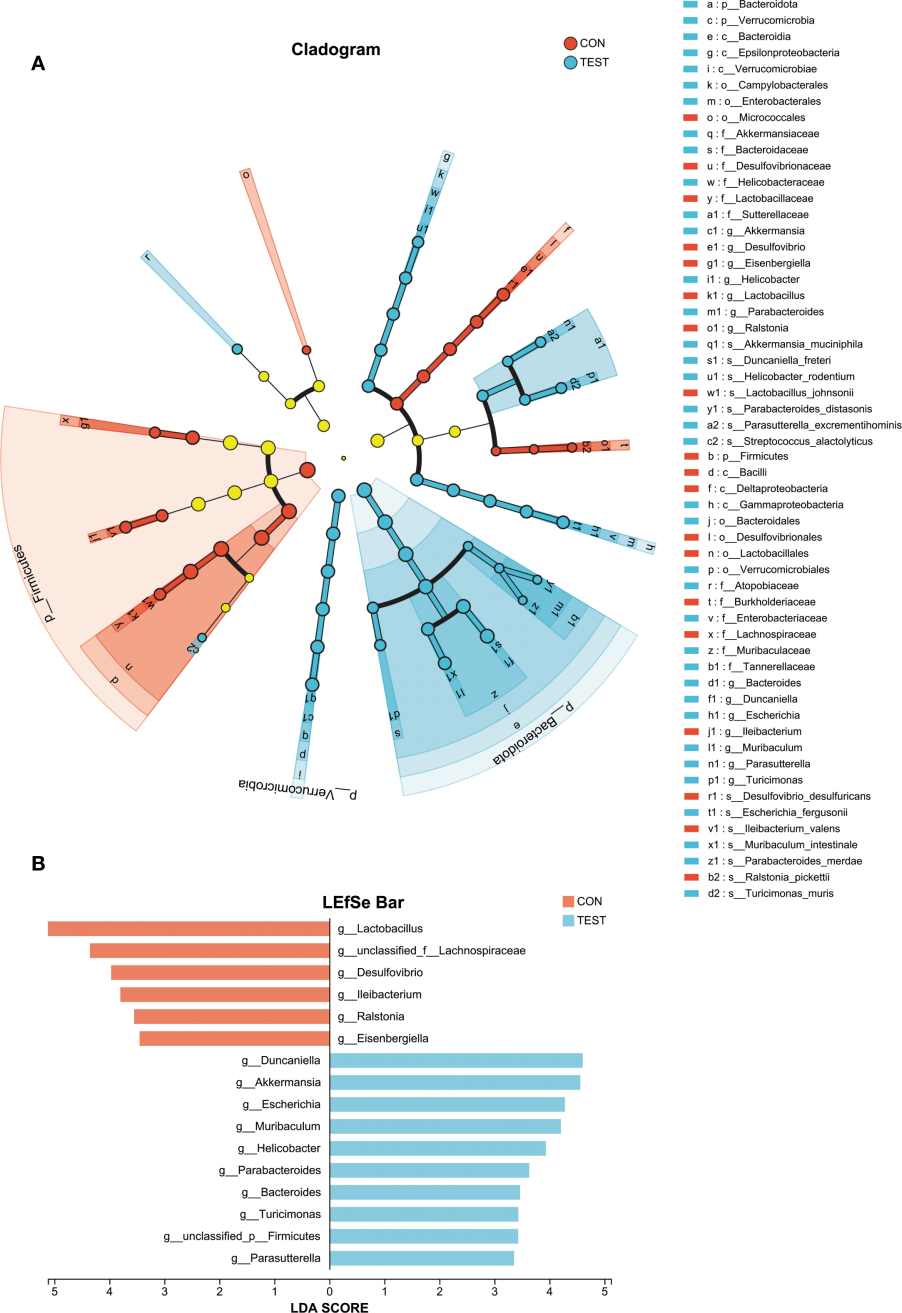
Analysis of species differences. Linear discriminant analysis (LDA) effect size (LEfSe) was used with LDA > 2, α = 0.05 (Kruskal-Wallis test with Benjamini-Hochberg correction) to analyze the differences between the control (CON) and the Metformin-treated (TEST) groups. **(A)** The phylogenetic tree shows species differences across taxonomic levels, highlighting microbial communities significantly enriched in specific groups (colored nodes) versus those with no significant difference/impact (yellow nodes). **(B)** The bar chart displays LDA scores indicating effect sizes for differentially abundant taxa.

To identify differentially abundant bacterial taxa, linear discriminant analysis effect size (LefSe) was applied with a logarithmic LDA score threshold of 2.0 and a significance level of α = 0.05. Taxonomic cladograms from phylum to species level are shown in **Figure 3A**. At the genus level, the control group was dominated by *Lactobacillus* in class *Firmicutes*; *Desulfovibrio* in class *Deltaproteobacteria; Eisenbergiella* and *unclassified_f:Lachnospiraceae* in class *Clostridia;* and *Ileibacterium* in class *Erysipelotrichia.* The Metformin-treated group exhibited higher abundances of *Parasutterella* and *Turicimonas* in class *Betaproteobacteria; Parabacteroides, Bacteroides, Duncaniella and Muribaculum in* class *Bacteroidia; Helicobacter* in class *Epsilonproteobacteria; Escherichia* in class *Gammaproteobacteria;* and *Akkermansia* in class *Verrucomicrobiae* (**Figures 3B** and **Supplementary Table S3**, LDA score > 2*, p*<0.05, Kruskal-Wallis test with Benjamini-Hochberg correction).

### Microbial functional prediction of gut microbiota in metformin-treated mice

**Figure 4:**
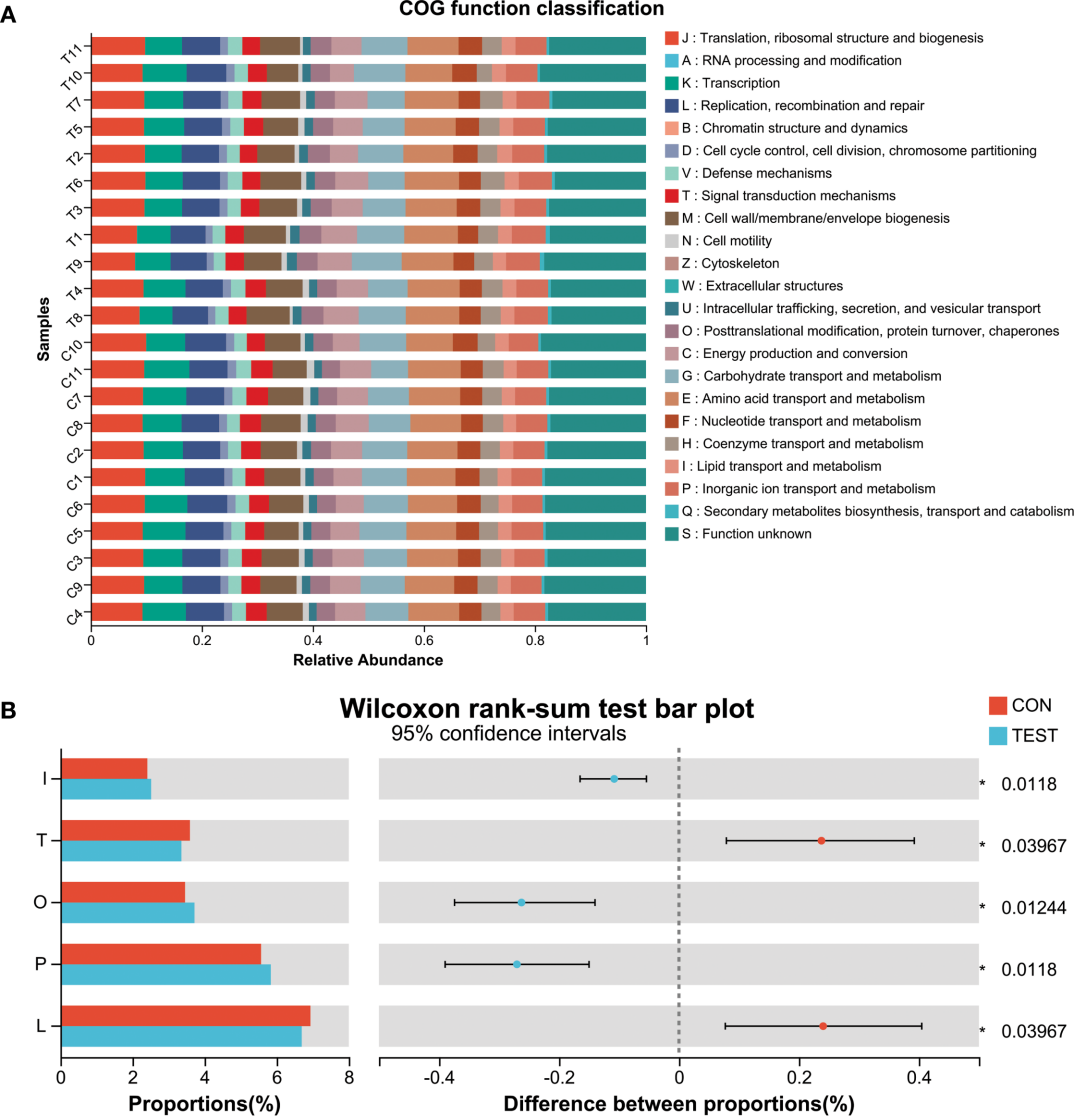
Microbial functional prediction of gut microbiota using PICRUSt2 analysis. **(A)** COG function classification. **(B)** Difference between groups. *adjusted *p*<0.05, Wilcoxon rank-sum test with Benjamini-Hochberg correction. L, Replication, recombination and repair; P, Inorganic ion transport and metabolism; O, Posttranslational modification, protein turnover, chaperones; T, Signal transduction mechanisms; I, Lipid transport and metabolism. CON, control group; TEST, Metformin-treated group.

To investigate the functional impact of metformin treatment on the gut microbiota of aged mice, Phylogenetic Investigation of Communities by Reconstruction of Unobserved States 2 (PICRUSt2) was employed to predict clusters of orthologous groups (COGs) and metabolic pathways using Kyoto Encyclopedia of Genes and Genomes (KEGG). Significant variations in COG functional categories were observed for Replication, recombination and repair; Inorganic ion transport and metabolism; Posttranslational modification, protein turnover, chaperones; Signal transduction mechanisms; Lipid transport and metabolism (adjusted *p*<0.05, Benjamini-Hochberg correction; **Figures 4A, B**).

**Figure 5:**
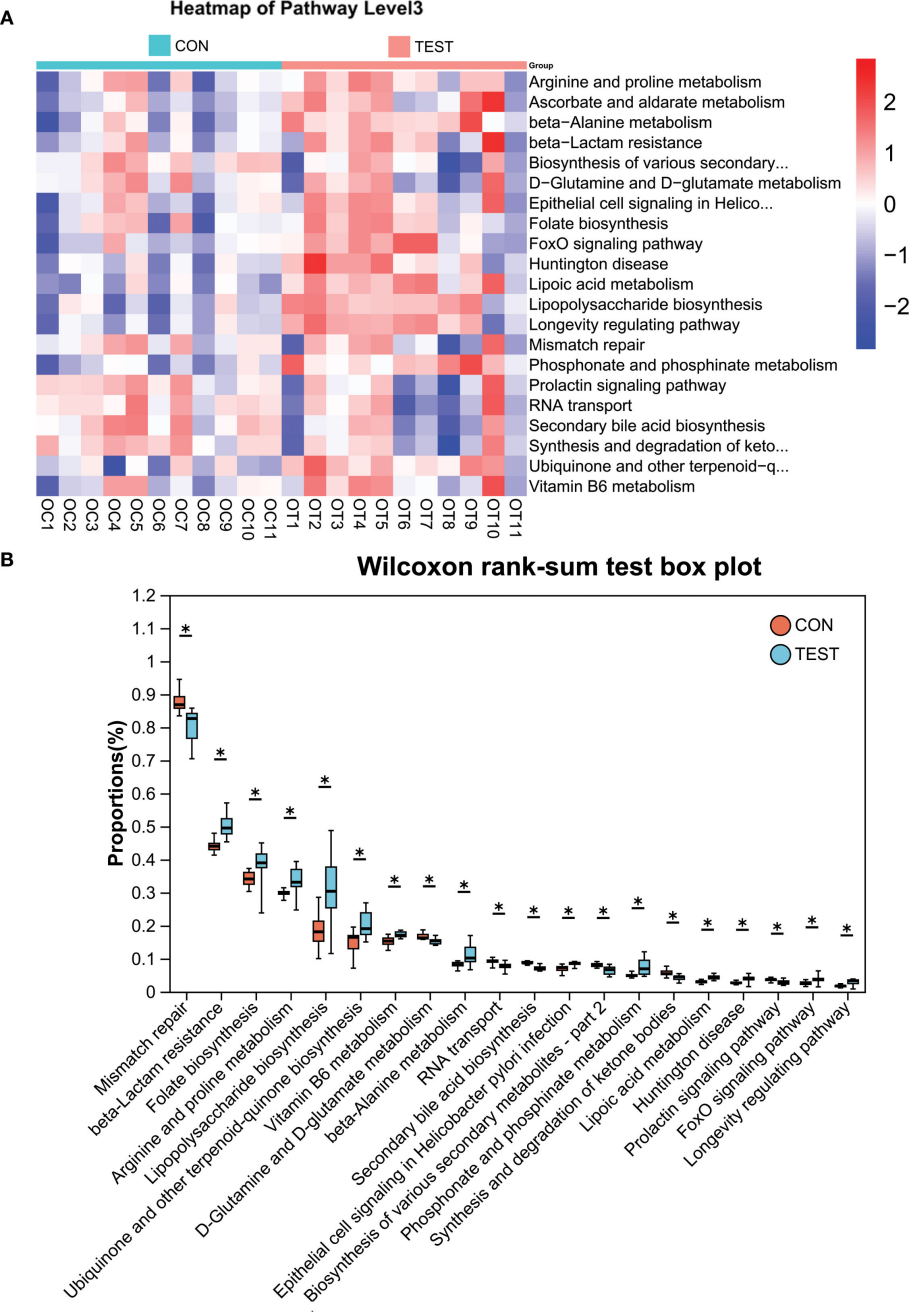
KEGG pathway analysis using PICRUSt2. **(A)** Heatmap of KEGG pathways (Level 3). **(B)** Differentially abundant pathways between groups. *adjusted *p*<0.05, Wilcoxon rank-sum test with Benjamini-Hochberg correction. CON, control group; TEST, Metformin-treated group.

To ensure clarity in distinguishing biologically relevant prokaryotic pathways from eukaryotic hits that may arise from database limitations, we systematically curated all significantly enriched KEGG pathways by cross-referencing them with established prokaryotic metabolic capabilities using MetaCyc’s bacterial pathway database. Pathways with functions exclusive to eukaryotic organisms were explicitly flagged as ‘non-bacterial’ in **Table 4** and excluded from biological interpretation. KEGG pathway analysis at Pathway Level 3 revealed significant alterations in microbial functionality, primarily associated with metabolism and biosynthesis, such as Secondary bile acid biosynthesis, Lipoic acid metabolism, beta-Lactam resistance, Mismatch repair, RNA transport, Lipopolysaccharide biosynthesis, and Phosphonate and phosphinate metabolism (adjusted *p*<0.05; **Figure 5A**). Among the top 30 differentially abundant pathways, notable differences were detected in metabolic and biosynthetic processes such as Secondary bile acid biosynthesis, Lipopolysaccharide biosynthesis, Vitamin B6 metabolism, beta-Alanine metabolism, and Arginine and proline metabolism (adjusted *p*<0.05; **Figure 5B**, **Supplementary Table S4**). Additionally, pathways potentially linked to aging, including Mismatch repair and Oxidative phosphorylation (30, 31), were enriched in the metformin-treated group (**Figure 5B**, **Supplementary Table S4**).

**Table 4:** Taxonomic distribution characteristics of KEGG signaling pathway.

KEGG pathway	Taxonomic distribution
Secondary bile acid biosynthesis	Cross-kingdom
Lipoic acid metabolism	Bacterial
Mismatch repair	Cross-kingdom
beta-Lactam resistance	Cross-kingdom
Terpenoid backbone biosynthesis	Non-bacterial
Carbon fixation in photosynthetic organisms	Cross-kingdom
D-Glutamine and D-glutamate metabolism	Cross-kingdom
Epithelial cell signaling in Helicobacter pylori infection	Non-bacterial
Biosynthesis of various secondary metabolites - part 2	Cross-kingdom
Huntington disease	Non-bacterial
Pentose phosphate pathway	Cross-kingdom
Vitamin B6 metabolism	Cross-kingdom
Monobactam biosynthesis	Cross-kingdom
RNA transport	Cross-kingdom
Longevity regulating pathway	Non-bacterial
Metabolic pathways	Cross-kingdom
Synthesis and degradation of ketone bodies	Non-bacterial
Folate biosynthesis	Cross-kingdom
Lipopolysaccharide biosynthesis	Cross-kingdom
Methane metabolism	Cross-kingdom
Ubiquinone and other terpenoid-quinone biosynthesis	Cross-kingdom
Phosphonate and phosphinate metabolism	Cross-kingdom
Carbon metabolism	Cross-kingdom
Ribosome	Cross-kingdom
Oxidative phosphorylation	Cross-kingdom
Glycine, serine and threonine metabolism	Cross-kingdom
Arginine and proline metabolism	Cross-kingdom
beta-Alanine metabolism	Cross-kingdom
Prolactin signaling pathway	Non-bacterial
FoxO signaling pathway	Non-bacterial

Bacterial, Bacterial signal pathway; Non-bacterial, Non-bacterial signaling pathway; Cross-kingdom, Both reported in prokaryotic and eukaryotic pathways.

Having established that metformin induces significant alterations in gut microbiota structure and metabolic potential, we next evaluated whether these microbial shifts associate with modulations in the splenic immune microenvironment.

### Effect of long-term metformin treatment on splenic immune cell populations in aged mice

**Figure 6:**
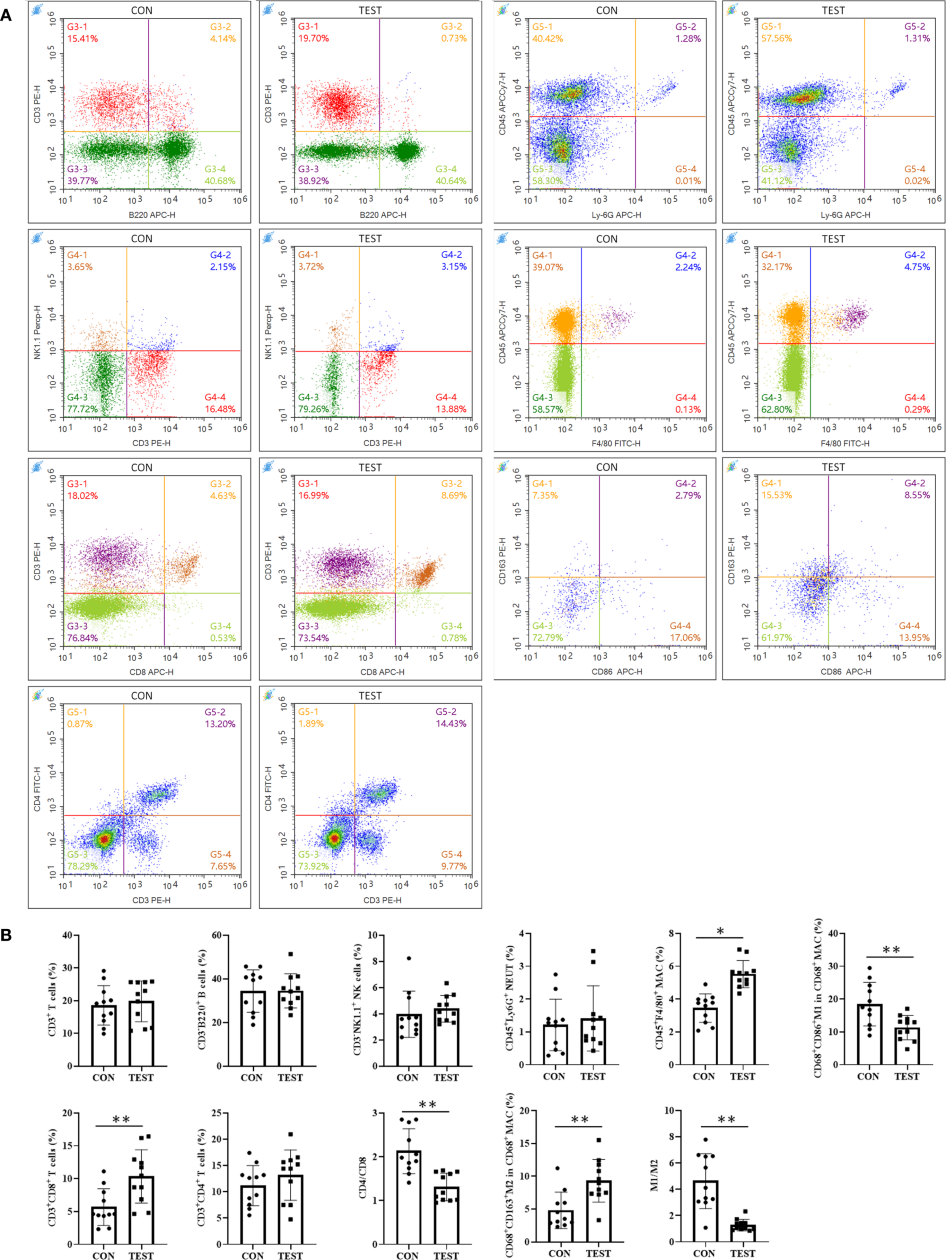
Effect of long-term metformin treatment on splenic immune cell populations in aged mice: FCM analysis. **(A)** Representative images of FCM; **(B)** Quantitative analysis of the indicated cells. Data represent the mean ± SD (n = 11). **p<0.01 (Student’s t-test). CON, control group; TEST, metformin-treated group.

To evaluate the impact of prolonged metformin treatment on splenic immune cell dynamics, FCM (**Figures 6A**, **Supplementary Figure S1**) was employed. Immune cell subsets were defined as follows: total T cells (CD3^+^), helper T cells (Th, CD3^+^CD4^+^), cytotoxic T cells (Tc, CD3^+^CD8^+^), B cells (CD3^-^B220^+^), natural killer cells (NK, CD3^-^NK1.1^+^), neutrophils (NEUT, CD45^+^Ly-6G^+^), macrophages (MAC, CD45^+^F4/80^+^), M1 macrophages (M1, CD68^+^CD86^+^), and M2 macrophages (M2, CD68^+^CD163^+^). Statistical analysis (**Figure 6B**) revealed that in the metformin-treated (TEST) group compared to control (CON), the percentage of Tc cells increased significantly from 5.67 ± 2.79% to 10.34 ± 4.06% (*p*<0.01, n = 11). Macrophage proportions also rose from 3.45 ± 0.87% to 5.52 ± 0.82% (*p*<0.05, n = 11). The Th/Tc (CD4/CD8) ratio decreased markedly (2.13 ± 0.51 vs. 1.31 ± 0.31; *p*<0.01, n = 11). Additionally, M1 macrophages decreased from 18.49 ± 6.23% to 11.33 ± 3.72% (*p*<0.01, n = 11), while M2 macrophages increased from 4.81 ± 2.77% to 9.32 ± 3.26% (*p*<0.01, n = 11). Consequently, the M1/M2 ratio declined from 4.63 ± 2.09 to 1.28 ± 0.44 (*p*<0.01, n = 11).

**Figure 7:**
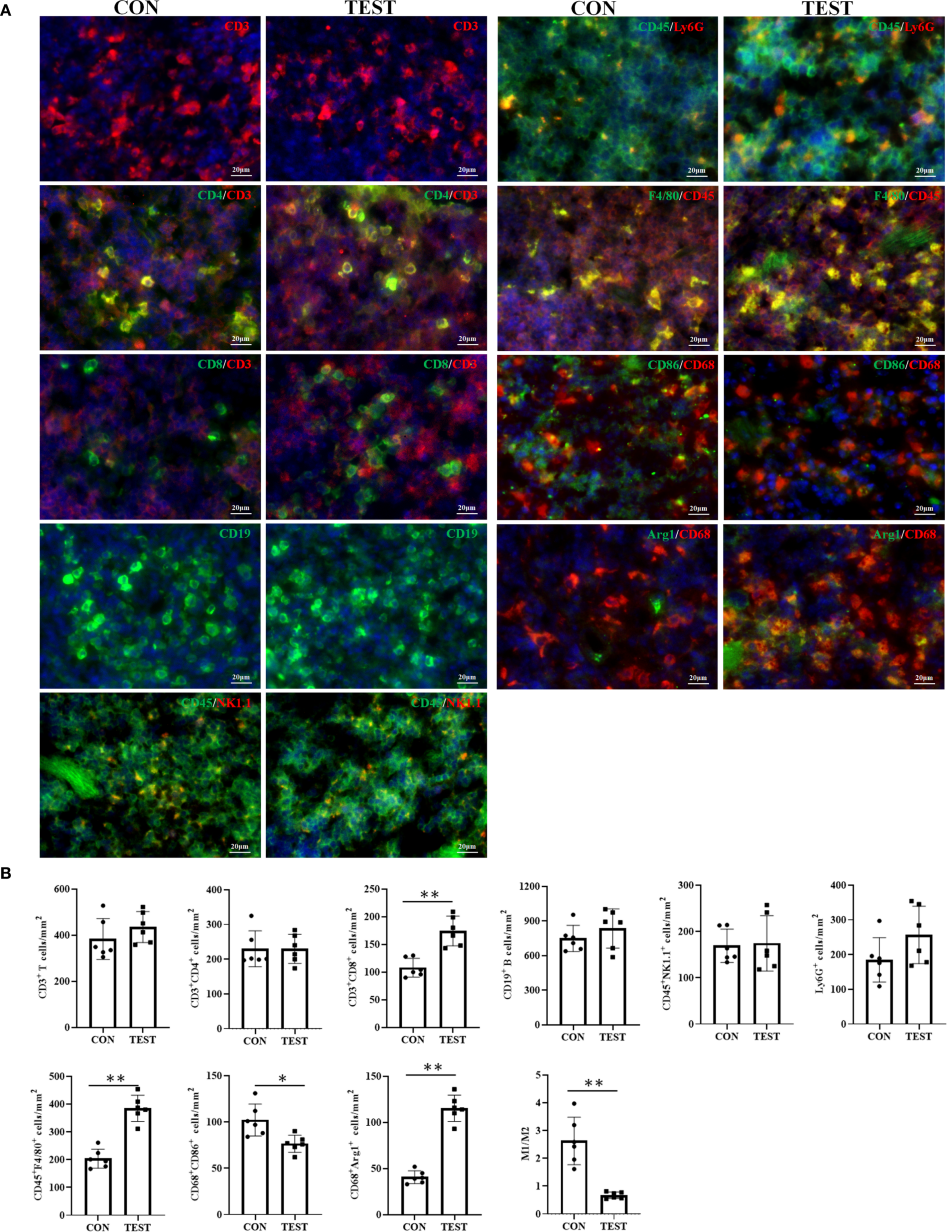
Effect of long-term metformin treatment on splenic immune cell populations in aged mice: IHF analysis. **(A)** Representative images of IHF; **(B)** Quantitative analysis of the indicated cells. Data represent the mean ± SD (n = 6). *p<0.05, **p<0.01 (Student’s t-test). CON, control group; TEST, metformin-treated group.

To further validate FCM results, immunohistochemical fluorescence (IHF) was performed. As shown in **Figure 7A**, CD3^+^ total T, CD3^+^CD4^+^ Th, CD3^+^CD8^+^ Tc, CD3^-^CD19^+^ B, CD3^-^NK1.1^+^ NK, CD45^+^Ly-6G^+^ NEUT, CD45^+^F4/80^+^ MAC, CD68^+^CCR7^+^ M1, and CD68^+^Arg1^+^ M2 were quantified in the CON and TEST groups. Statistical analysis (**Figure 7B**) indicated that in the CON group, cell densities (cells/mm²) were as follows: T cells (383.33 ± 88.65), Th cells (230.00 ± 51.72), Tc cells (108.00 ± 16.92), B cells (748.33 ± 113.70), NK cells (168.92 ± 35.85), NEUT (184.83 ± 63.85), MAC (203.83 ± 34.38), M1 (102.17 ± 17.24), and M2 (40.67 ± 6.95). In the TEST group, these values were 435.33 ± 66.95 (T cells), 230.00 ± 41.84 (Th cells), 174.17 ± 26.78 (Tc cells), 835.00 ± 169.64 (B cells), 174.50 ± 59.74 (NK cells), 256.67 ± 82.66 (NEUT), 384.67 ± 47.26 (MAC), 76.50 ± 9.22 (M1), and 115.33 ± 14.25 (M2). Comparisons between groups demonstrated significant increases in Tc cells, macrophages, and M2 macrophages in the TEST group (*p*<0.01, n = 6), while M1 macrophage numbers and the M1/M2 ratio were reduced (*p*<0.05 or 0.01, n = 6). No significant differences were observed in other cell populations (*p* > 0.05, n = 6).

### Effects of long-term metformin treatment on the mRNA expression of immune cell subsets in aged mouse spleen

**Figure 8:**
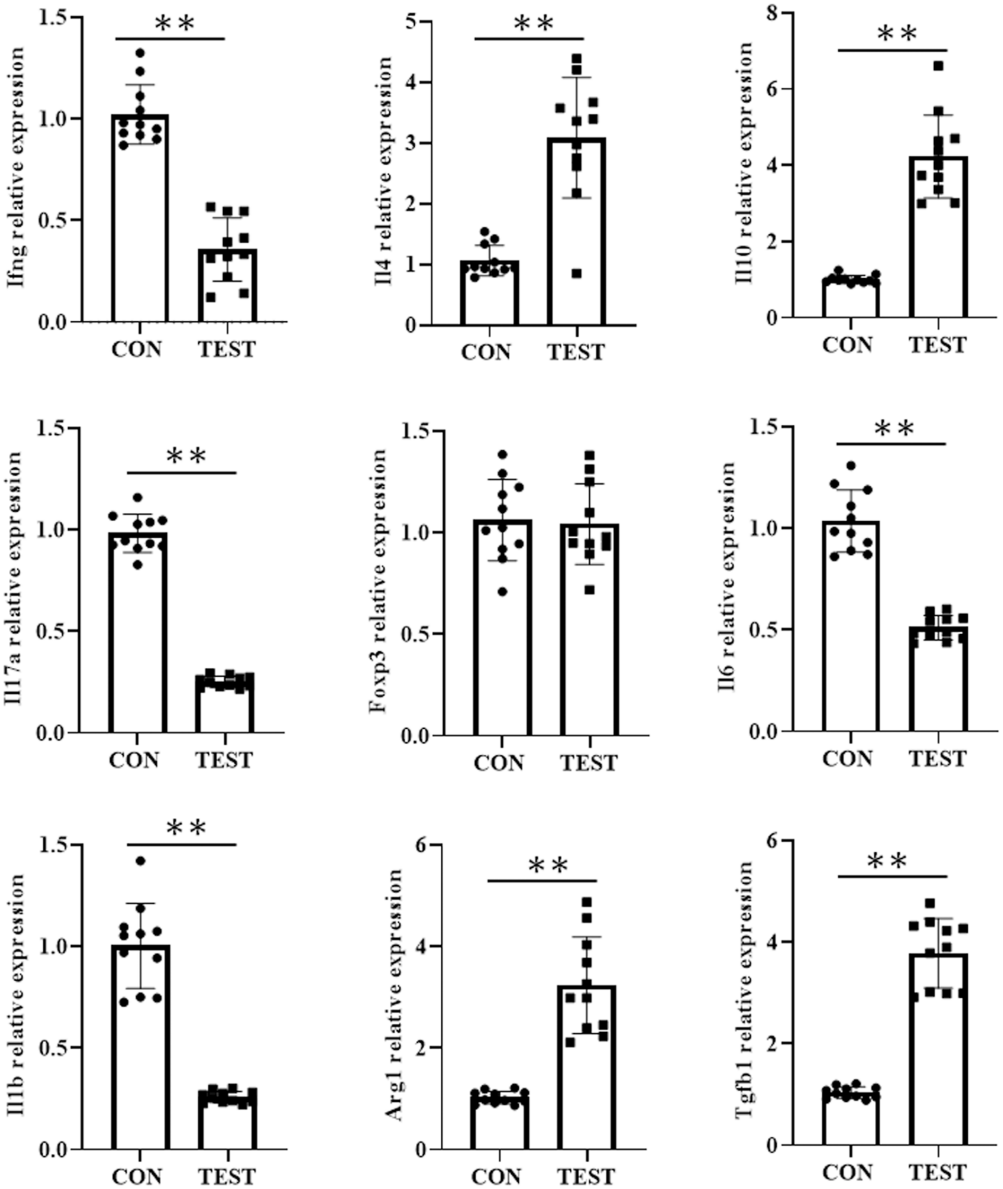
Effect of long-term metformin treatment on the mRNA expression in aged mouse spleen. Quantitative analysis of the indicated mRNA expression in control group (CON) and metformin-treated group (TEST). Data represent the mean ± SD (n = 11). **p<0.01 (Student’s t-test).

To further investigate the impact of long-term metformin treatment on immune cell differentiation in the spleen of aged mice, reverse transcription quantitative polymerase chain reaction (RT-qPCR) was used to assess mRNA levels of Th1-, Th2-, Th17-, Treg-, M1-, and M2-associated cytokines or markers. As shown in **Figure 8**, the mRNA levels of cytokines or markers of Th1 (*Infg*), Th17 (*Il17a*), and M1 (*I11b* and *Il6*) were significantly higher in CON group compared to the TEST group (all *p*<0.01, n = 11). In contrast, mRNA levels of Th2 (*Il4* and *Il10*), and M2 (*Arg1* and *Tgfb1*) were markedly reduced in the TEST group (all *p*<0.01, n = 11). Notably, no significant difference was observed in *Foxp3* expression, a Treg-specific marker, between the two groups (*p* > 0.05, n = 11).

### Correlation between gut microbiota composition and splenic immune cell profiles in aged mice

**Figure 9:**
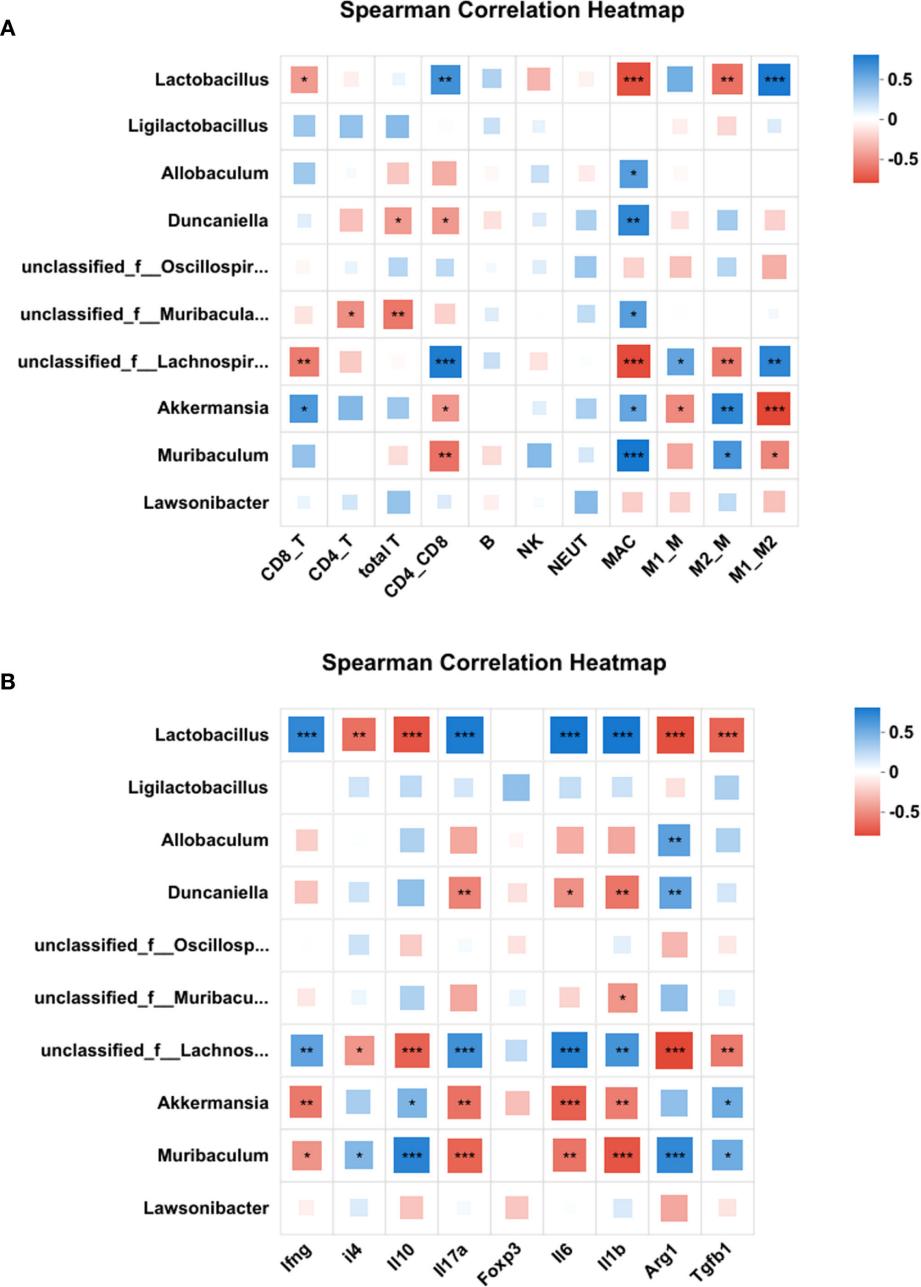
Correlation between gut microbiota composition and splenic immune cell profiles. Heatmaps of the correlation between the top 10 bacterial genera at the genus level and the immune cell subsets **(A)** or cytokine/marker mRNA levels **(B)**. Correlations were quantified by Spearman’s ρ and tested for significance (Benjamini-Hochberg FDR-adjusted *p*<0.05). Color intensity reflects correlation strength and direction. Red squares represent a negative correlation, while blue squares represent a positive correlation. *p<0.05, **p<0.01, ***p<0.001. CD8_T, cytotoxic T cells; CD4_T, helper T cells; CD4_CD8, CD4/CD8 ratio;NK, Natural killer cells; NEUT, Neutrophil; MAC, macrophages; M1_M, M1 macrophages; M2_M, M2 macrophages; M1_M2, M1/M2 ratio.

To investigate the relationship between splenic immune cell subsets and gut microbial composition in aged mice, we analyzed the correlation between the relative abundance of dominant gut bacterial genera and immune cell subsets in the spleen. As illustrated in **Figure 9**, the top 10 bacterial genera at the genus level showed distinct correlation patterns with immune parameters.

As shown in **Figure 9A** and **Supplementary Table S5**, *Lactobacillus* exhibited significant negative correlations with Tc cells (cytotoxic T cells), macrophages (MAC), and M2 macrophages, while showing positive correlations with the CD4/CD8 ratio and M1/M2 ratio. *Allobaculum* was exclusively positively correlated with macrophages. *Duncaniella* negatively correlated with total T cells and CD4/CD8 ratio, but positively with MAC. *Unclassified Muribaculaceae* negatively associated with total T cells and Th cells (helper T cells), yet positively with MAC. *Unclassified Lachnospiraceae* demonstrated negative correlations with Tc cells, MAC, and M2 macrophages, but positive correlations with CD4/CD8 ratio, M1 macrophages, and M1/M2 ratio. *Akkermansia* and *Muribaculum* showed reciprocal correlation patterns: *Akkermansia* negatively correlated with CD4/CD8 ratio, M1 macrophages, and M1/M2 ratio, but positively with Tc cells, MAC, and M2 macrophages; *Muribaculum* showed negative correlations with CD4/CD8 ratio and M1/M2 ratio, but positive correlations with MAC and M2 macrophages.

Furthermore, correlations between microbial abundance and cytokine/marker mRNA levels were analyzed. As shown in **Figure 9B** and **Supplementary Table S6**, *Lactobacillus* and *Unclassified Lachnospiraceae* were negatively associated with anti-inflammatory markers (*Il4, Il10, Arg1, and Tgfb1*) and positively correlated with pro-inflammatory cytokines (*Ifng, Il17a, Il6, and Il1b*). Conversely, *Akkermansia* and *Muribaculum* showed opposite trends, positively correlating with anti-inflammatory markers (*Il4, Il10, Arg1, and Tgfb1*) and negatively with pro-inflammatory cytokines (*Ifng, Il17a, Il6, and Il1b*). *Duncaniella* and *Unclassified Muribaculaceae* exhibited mixed profiles: *Duncaniella* negatively associated with *Il17a, Il6*, and *Il1b* but positively with *Arg1*, while *Unclassified Muribaculaceae* only showed a negative correlation with *Il1b*.

These correlation results suggest that higher abundances of *Lactobacillus* and *unclassified Lachnospiraceae* are associated with a pro-inflammatory state, whereas *Akkermansia* and *Muribaculum* are linked to anti-inflammatory responses.

## Discussion

This study provides compelling evidence that long-term metformin administration ameliorates age-related splenic immune modulation in mice, primarily through gut microbiota modulation. By integrating microbiome and immune profiling, we delineate a tripartite relationship between metformin, gut microbial communities, and splenic immune microenvironment, offering novel insights into the gut-spleen axis in immunosenescence.

Our data reveal that metformin counteracts these changes by enhancing cytotoxic T cell (Tc) and macrophage populations while suppressing pro-inflammatory M1 polarization. The increased Tc proportion aligns with prior studies showing metformin’s ability to augment CD8^+^ T cell responses in cancer and infection models, potentially via AMPK-mediated metabolic reprogramming (32, 33). Concurrently, the shift from M1 to M2 macrophages underscores metformin’s anti-inflammatory role, consistent with its known inhibition of NLRP3 inflammasome activation (34). Together, these findings highlight metformin’s capacity to enhance adaptive immunity (via Tc expansion) and resolve inflammation (via macrophage repolarization) in the aged spleen.

A key novelty of this study lies in linking metformin-induced gut microbiota changes to splenic immune remodeling. The enrichment of *Akkermansia muciniphila*, a mucin-degrading bacterium associated with improved gut barrier integrity, correlates with reduced systemic inflammation and enhanced SCFA production, which may promote T cell differentiation and macrophage homeostasis (35). Conversely, the proportion reduction of *Lactobacillus*, which is traditionally viewed as a beneficial genus (36), was unexpected in our model. This might indicate strain-specific effects or context-dependency, potentially explaining the observed negative correlation with anti-inflammatory markers and association with Th17 responses in aged hosts. The rise in *Muribaculum* and *Duncaniella*, both linked to carbohydrate metabolism (37, 38), aligns with metformin’s ability to enhance microbial butyrate synthesis, a known regulator of Treg/Th17 balance (39, 40). Notably, the divergent correlations between microbial taxa and immune parameters (e.g., *Akkermansia* with anti-inflammatory cytokines vs. *Lactobacillus* with pro-inflammatory markers) suggest complex taxon-specific roles in immunosenescence. This dichotomy could potentially arise from functional redundancy within microbial communities or context-dependent interactions with host immunity.

Importantly, the key microbial shifts observed in our aged mouse model following metformin treatment—specifically the enrichment of *Akkermansia* and *Escherichia*, alongside the reduction in certain *Lactobacillus* species—resonate strongly with findings from human metformin studies. For instance, large-scale integrated analyses of human gut metagenomes consistently report metformin-induced increases in *Akkermansia muciniphila* abundance across diverse populations (41–43). For instance, metformin consistently increases *Akkermansia muciniphila* abundance in humans, aligning with our correlation between *Akkermansia* enrichment and anti-inflammatory splenic M2 macrophages. Similarly, the rise in *Escherichia* noted in our mice mirrors observations in metformin-treated T2D patients, where increased *Escherichia coli* abundance has been mechanistically linked to gastrointestinal side effects but also potentially to microbial agmatine production (42, 44). Recent human studies further indicate that metformin’s inhibition of the microbial enzyme agmatinase elevates agmatine levels (44), a metabolite implicated in enhancing host fatty acid oxidation—a process potentially underpinning the Tc cell metabolic fitness observed in our splenic phenotype. Moreover, akin to our correlation linking *Muribaculum* to improved metabolic indices, human studies show metformin enriches mucin-degrading and SCFA-producing taxa (including related members of the *Muribaculaceae* family), contributing to improved glucose homeostasis and immune modulation (41, 45). These conserved microbial signatures across species, with increases in *Akkermansia* and *Escherichia* and a decrease in *Lactobacillus*, underscore the translatability of gut microbiota-mediated mechanisms in metformin’s immunometabolic actions, reinforcing the relevance of our murine model to human pathophysiology.

PICRUSt2-predicted functional alterations further support a microbiota-driven mechanism. The enrichment of oxidative phosphorylation pathways mirrors metformin’s mitochondrial effects (46), suggesting a symbiotic relationship between host and microbial metabolism. Enhanced bile acid metabolism, particularly involving SBAs, functions as a critical communication pathway between the gut microbiota and the host (47). SBAs like deoxycholic acid (DCA) and lithocholic acid (LCA), generated via microbial biotransformation, act as potent signaling molecules activating host receptors (FXR, TGR5) expressed in systemic tissues including the spleen liver, and brain (48–50). TGR5 activation triggers cAMP-PKA signaling, promoting NLRP3 inflammasome degradation and suppressing IL-1β release (51, 52). For example, microbial DCA alleviates inflammation via TGR5-cAMP-PKA-NLRP3 pathways (53), while impaired TGR5 exacerbates inflammation (54). Thus, SBAs serve as essential microbial-host messengers, bridging gut microbiota activity with systemic immunity through FXR/TGR5-dependent NLRP3 regulation (55–57). This axis represents a key mechanism for immune-metabolic balance. Conversely, downregulation of mismatch repair pathways might reflect reduced genomic instability (58).

Our findings complement multi-omics studies linking gut microbiota shifts to systemic aging outcomes (59–63). While prior work focused on liver, brain, or metabolism, our study specifically maps metformin-induced microbial remodeling (enrichment of *Akkermansia, Muribaculum*; reduction of *Lactobacillus*) to a defined splenic immune phenotype (enhanced Tc, M2 polarization) within aging. Furthermore, metformin directly reprograms bacterial metabolism via the phosphotransferase system (PTS) and Crp, leading to agmatine accumulation (64). Bacterium-derived agmatine is essential for metformin’s induction of host FAO (64)—a metabolic shift regulated by factors like NHR-49/PPARα that provides energy and signaling molecules potentially driving the observed splenic immunophenotypes, including increased Tc activity and anti-inflammatory macrophage polarization (64–66).

While our findings align with metformin’s documented anti-inflammatory properties, some observations diverge from earlier studies. For example, metformin’s failure to upregulate *Foxp3* (a Treg marker) contrasts with its reported induction of Tregs in adipose tissue (67), potentially due to tissue-specific epigenetic regulation or differential microbiota in aged vs. obese models. Similarly, the lack of change in B cell populations contradicts metformin’s reported enhancement of humoral immunity in vaccination models (19). This discrepancy likely stems from fundamental age-related B cell alterations: immunosenescence involves reduced naïve B cell output, accumulation of exhausted/age-associated B cells (ABCs), impaired germinal center formation, and diminished antigen responsiveness (68, 69)—all consistent with our baseline immunosenescent phenotype. Metformin’s documented B cell effects occur predominantly in young/adult models with intact B cell receptor signaling and functional Tfh cells, mechanisms compromised in aging (70, 71). Furthermore, our correlation analysis revealed no significant links between metformin-altered taxa and splenic B cells (**Figure 9**), suggesting the microbiome-immune axis in aging may preferentially modulate T cell/macrophage pathways.

Critically, our findings suggest that metformin’s splenic immunomodulation may be mediated through the gut-spleen axis. The enrichment of *Akkermansia* and *Muribaculum* correlates with increased anti-inflammatory M2-macrophages, aligning with evidence that *Akkermansia*-derived extracellular vesicles (EVs) enter circulation, directly modulating splenic immune cells (72, 73). Reduced *Lactobacillus* abundance might modulate splenic immunity through alterations in bile acid metabolism (74, 75). Metformin inhibits bile acid reabsorption, which increases distal gut bile acids (76, 77) and elevates circulating conjugated bile acids. These bile acids act as FXR antagonists to suppress splenic Th17 differentiation (78, 79).

The gut-spleen crosstalk may explain the *Lachnospiraceae* reduction despite its butyrogenic potential. Metformin suppresses some butyrate producers (e.g., *Fecalibacterium*) yet enriches others like *Duncaniella* (39), potentially favoring acetate production. Acetate can enhance splenic Tc cytotoxicity via histone deacetylase inhibition (45, 80). Additionally, metformin’s inhibition of microbial agmatinase elevates agmatine (44), a metabolite suppressing macrophage polarization and T cell responses (81, 82). Although correlative data alone cannot definitively establish functional crosstalk, our integrated dataset supports this hypothesis.

Consequently, this study has several limitations warrant consideration. First, while our correlation analyses and functional predictions suggest that metformin-induced microbiota alterations may contribute to splenic immune remodeling, it is important to emphasize that these associations do not establish causality. Although FMT studies in diabetic models have established a causal role for the microbiota in mediating metformin’s metabolic effects (83), its role in immune aging remains unexplored. Future studies should employ fecal microbiota transplantation (FMT) from metformin-treated aged mice into untreated counterparts or germ-free recipients. These experiments are essential to directly determine if microbiota transfer recapitulates the observed immune benefits. Additionally, integrating metabolite profiling (e.g., of SCFAs and bile acids) in follow-up studies is recommended to identify the mediators linking microbial changes to splenic immunity.

Second, the study’s exclusive use of male mice is a recognized limitation, as sexual dimorphism influences both gut microbiota composition and immune aging trajectories. Therefore, our findings may not generalize to females. Future work should include female cohorts to evaluate sex-specific effects of metformin on the gut-spleen axis, particularly given hormonal impacts on immunometabolism.

Third, mechanisms linking specific taxa (e.g., *Akkermansia*) to splenic Tc cells remain unclear. Single-cell RNA sequencing of gut-derived immune cells could elucidate migratory patterns and transcriptional programs.

Fourth, this study exclusively utilized aged mice as both treatment and control groups, precluding direct comparisons with young adult immune and microbial profiles. While this design robustly demonstrates metformin’s effects within an aging context, it cannot delineate whether observed improvements represent restoration toward a youthful state or establishment of a novel compensatory equilibrium. We mitigated this constraint by contextualizing our aged control data against established benchmarks for murine immunosenescence and age-related dysbiosis from seminal literature (12, 84–86). Nevertheless, future investigations should incorporate young adult controls to definitively ascertain metformin’s capacity to reverse—rather than merely attenuate—aging-associated decline.

Fifth, although we documented metformin-induced changes in immune cell frequencies and cytokine profiles, we did not functionally challenge the immune system through vaccination or pathogen exposure. Thus, it remains unknown whether the remodeling of the splenic immune landscape translates to enhanced functional immunity, such as improved antibody responses or pathogen clearance. Future studies incorporating such functional assays will be crucial to fully ascertain the physiological relevance of metformin-mediated immunomodulation in aging.

Sixth, immunosenescence involves a coordinated decline in both central (thymic) and peripheral immunity (87). Our study focused on the spleen and thus did not evaluate the potential impact of metformin on thymic integrity or naïve T cell egress. Therefore, it remains unclear whether the expansion of splenic cytotoxic T cells originates from enhanced thymopoiesis, peripheral expansion, or altered survival. Future investigations including analysis of thymic architecture, T-cell receptor excision circles (TRECs) (88), and detailed phenotyping of recent thymic emigrants (89) would help delineate the relative contributions of central versus peripheral mechanisms.

Seventh, our findings are specific to the splenic immune microenvironment. Given the anatomical and functional specialization of lymphoid organs (90, 91), metformin’s effects may differ in lymph nodes (e.g., mesenteric vs. peripheral) or gut-associated lymphoid tissue (GALT). Whether the microbiota-driven immunomodulation we report is confined to the spleen or represents a broader systemic effect remains to be determined. Future comparative analyses of multiple lymphoid sites will be essential to map the full anatomical scope of the gut–immune axis influenced by metformin.

Eighth, in line with the observed changes in total T cell populations, a key limitation is the lack of high-resolution phenotyping of T cell differentiation states. Immunosenescence entails not only changes in total CD4^+^ or CD8^+^ T cell numbers but also a fundamental shift in subset composition—specifically, the attrition of naïve T cells (Tn) and the accumulation of memory and senescent-like effector cells (1, 92). Without analyzing markers, such as CD45RA, CD44, CCR7 and CD62L, to distinguish Tn, central memory (Tcm), effector memory (Tem), and terminally differentiated effector (Temra) subsets (93–95), we cannot definitively characterize the metformin-expanded Tc population. Future multi-parameter flow cytometric analyses are necessary to determine whether this expansion reflects rejuvenation of the naïve repertoire, preferential expansion of a memory subset, or altered T cell survival.

Finally, translating these findings to humans requires validation in elderly cohorts, particularly given inter-species microbiota differences.

From a translational perspective, our data position metformin as a dual-purpose agent targeting metabolic and immune aging. The gut microbiota shifts, particularly *Akkermansia* enrichment, resemble calorie restriction (CR) effects (96), suggesting metformin may mimic CR pharmacologically.

The gut-spleen axis opens avenues for combinational therapies, such as metformin with probiotics (e.g., *Akkermansia muciniphila* formulations) or prebiotics targeting *Muribaculum*-associated pathways, to synergistically enhance immune resilience in the elderly. This could allow dose reduction to minimize side effects. The axis also provides a biomarker framework (e.g., circulating microbial metabolites, splenic immune cell profiles via imaging) for monitoring efficacy.

However, important translational challenges must be acknowledged. Murine models exhibit fundamental differences from humans in gut microbiota composition, immune aging patterns, and metformin pharmacokinetics. These interspecies disparities necessitate caution when extrapolating our findings to human aging. Refer to the method of dose conversion between experimental animals and humans in preclinical and clinical stages of drug development (97), the metformin dose used (300 mg/kg/day) translates to a human equivalent dose (HED) of ~24.3 mg/kg (~1,460 mg/day for 60kg adult), within the standard clinical range, supporting pharmacological relevance but necessitating human validation.

To bridge the translational gap, we propose: 1) Longitudinal metformin trials in elderly with paired fecal metagenomics and immune profiling; 2) Correlative analyses of existing cohorts (e.g., NHANES) examining metformin use, gut microbiota signatures, and age-related immune markers; and 3) FMT studies from metformin-treated elderly donors to germ-free mice to validate causal microbiota-immune relationships. Such approaches would help determine whether the gut-spleen axis observed here is conserved in human aging and whether microbiota-directed signatures could serve as biomarkers for metformin’s geroprotective efficacy.

## Conclusion

In summary, this study demonstrates that metformin reshapes the gut microbiota, which is associated with mitigation of age-associated splenic immune dysregulation, favoring anti-inflammatory macrophage polarization and cytotoxic T cell expansion. These findings establish the gut-spleen axis as a novel therapeutic target and position metformin as a promising microbiota-directed geroprotective agent. Future research should prioritize mechanistic dissection of gut-spleen communication and clinical validation of metformin’s geroprotective efficacy in human populations.

## Data Availability

The datasets presented in this study can be found in online repositories. The names of the repository/repositories and accession number(s) can be found below: https://www.ncbi.nlm.nih.gov/sra/PRJNA1259763.

## Supplementary material

Supplementary Figure 1 Gating strategy for identification of immune cell populations by FCM. **(A)** Splenic immune cells were gated based on FSC-A/SSC-A, followed by selection of single cells using FSC-A/FSC-H. T cells were identified as CD3^+^, with further subdivision into CD4^+^ and CD8^+^ subsets. **(B)** B cells (CD3^-^B220^+^) and NK cells (CD3^-^NK1.1^+^) were identified among CD3^-^ lymphoid cells. **(C)** CD45^+^Ly-6G^+^ population was defined as neutrophils and CD45^+^F4/80^+^ as macrophages. **(D)** CD68^+^ macrophages were further classified into M1 (CD68^+^CD86^+^) and M2 (CD68^+^CD163^+^) phenotypes. Fluorescence thresholds were established using isotype-matched control antibodies.

Supplementary Figure 2 Metformin treatment increases microbial diversity. Alpha diversity indices comparing control group (CON) and metformin-treated group (TEST). **(A)** Chao1 index (assessing sequencing depth), showing no significant difference (*p* > 0.05), Wilcoxon rank-sum test (n = 11). **(B)** Shannon index (assessing species diversity), showing a significant increase in the metformin group (**p*<0.05), Wilcoxon rank-sum test (n = 11).

Supplementary Figure 3 Differential genus abundance (ANCOM-BC2) and relative abundance heatmap. CON, control group; TEST, metformin-treated group.

Supplementary Table 1 The post-processed 16S-count at ASV-level.

Supplementary Table 2 The post-processed 16S-count at genus-level.

Supplementary Table 3 LEfSe analysis identifies bacterial taxa with differential abundance between CON and TEST groups. The table lists taxonomic units from phylum to genus level with a Linear Discriminant Analysis (LDA) score > 2.0 and a Benjamini-Hochberg corrected p-value < 0.05 (Kruskal-Wallis test). The group column indicates the group (CON or TEST) in which the taxon is significantly enriched. The Mean column represents the average relative abundance (log-transformed), the LDA value indicates the effect size (magnitude of the difference), and the P_value is the corrected significance value. CON, control group; TEST, metformin-treated group.

Supplementary Table 4 Differential Abundance of predicted KEGG pathways between TEST and CON groups. Predictions are based on KEGG pathway abundances derived from the COG functional annotations within PICRUSt2. Calculated as CON-Mean(%)/TEST-Mean(%). Values > 1 indicate higher predicted abundance in the CON group; values < 1 indicate higher predicted abundance in the TEST group. Statistical Analysis includes 95% confidence intervals (Lower ci, Upper ci), effect size (Effect size), raw p-value (P-value), and corrected p-value to account for multiple hypothesis testing. Pathways are typically considered significantly different if corrected p-value < 0.05. CON, control group; TEST, metformin-treated group.

Supplementary Table 5 Spearman’s correlation between gut microbiota genera and immune cell profiles. This table presents Spearman’s rank correlation coefficients (ρ) and Benjamini-Hochberg-adjusted p-values for associations between gut microbiota genera (rows) and immune cell populations (columns).

Supplementary Table 6 Spearman’s correlation between gut microbiota genera and cytokine/marker mRNA levels. This table presents Spearman’s rank correlation coefficients (ρ) and Benjamini-Hochberg-adjusted p-values for associations between gut microbiota genera (rows) and cytokine/marker mRNA levels (columns).

## References

[1] Liu Z Liang Q Ren Y Guo C Ge X Wang L. Immunosenescence: molecular mechanisms and diseases. Signal Transduct Target Ther. (2023) 8:200. doi: 10.1038/s41392-023-01451-2, PMID: 37179335 PMC10182360

[2] Chen R Zou J Chen J Wang L Kang R Tang D. Immune aging and infectious diseases. Chin Med J (Engl). (2024) 137:3010–49. doi: 10.1097/CM9.0000000000003410, PMID: 39679477 PMC11706578

[3] Quiros-Roldan E Sottini A Natali PG Imberti L. The impact of immune system aging on infectious diseases. Microorganisms. (2024) 12:775. doi: 10.3390/microorganisms12040775, PMID: 38674719 PMC11051847

[4] Liu Z Zuo L Zhou Z Liu S Ba Y Zuo A. Targeting immunosenescence for improved tumor immunotherapy. MedComm. (2020) . 2024 5:e777. doi: 10.1002/mco2.777, PMID: 39473905 PMC11518697

[5] Picker LJ Butcher EC. Physiological and molecular mechanisms of lymphocyte homing. Annu Rev Immunol. (1992) 10:561–91. doi: 10.1146/annurev.iy.10.040192.003021, PMID: 1590996

[6] Turner VM Mabbott NA. Influence of ageing on the microarchitecture of the spleen and lymph nodes. Biogerontology. (2017) 18:723–38. doi: 10.1007/s10522-017-9707-7, PMID: 28501894 PMC5597693

[7] Nikolich-Zugich J Davies JS. Homeostatic migration and distribution of innate immune cells in primary and secondary lymphoid organs with ageing. Clin Exp Immunol. (2017) 187:337–44. doi: 10.1111/cei.12920, PMID: 28035684 PMC5290228

[8] Masters AR Haynes L Su DM Palmer DB. Immune senescence: significance of the stromal microenvironment. Clin Exp Immunol. (2017) 187:6–15. doi: 10.1111/cei.12851, PMID: 27529161 PMC5167042

[9] Jiang B Dong YN Xiong Y Jiang CX Ping J Wu Q. Global research trends in inflammaging from 2005 to 2024: a bibliometric analysis. Front Aging. (2025) 6:1554186. doi: 10.3389/fragi.2025.1554186, PMID: 40276724 PMC12018403

[10] Winkler ES Thackray LB. A long-distance relationship: the commensal gut microbiota and systemic viruses. Curr Opin Virol. (2019) 37:44–51. doi: 10.1016/j.coviro.2019.05.009, PMID: 31226645 PMC6768733

[11] Palm NW de Zoete MR Flavell RA. Immune-microbiota interactions in health and disease. Clin Immunol. (2015) 159:122–7. doi: 10.1016/j.clim.2015.05.014, PMID: 26141651 PMC4943041

[12] Bosco N Noti M. The aging gut microbiome and its impact on host immunity. Genes Immun. (2021) 22:289–303. doi: 10.1038/s41435-021-00126-8, PMID: 33875817 PMC8054695

[13] Shin HK Bang YJ. Aromatic amino acid metabolites: molecular messengers bridging immune-microbiota communication. Immune Netw. (2025) 25:e10. doi: 10.4110/in.2025.25.e10, PMID: 40078785 PMC11896664

[14] Hu Y Aljumaah MR Azcarate-Peril MA. Galacto-oligosaccharides and the elderly gut: implications for immune restoration and health. Adv Nutr. (2024) 15:100263. doi: 10.1016/j.advnut.2024.100263, PMID: 38897384 PMC11292246

[15] Kavanagh K Hsu FC Davis AT Kritchevsky SB Rejeski WJ Kim S. Biomarkers of leaky gut are related to inflammation and reduced physical function in older adults with cardiometabolic disease and mobility limitations. Geroscience. (2019) 41:923–33. doi: 10.1007/s11357-019-00112-z, PMID: 31654268 PMC6925090

[16] Sun CM Hall JA Blank RB Bouladoux N Oukka M Mora JR. Small intestine lamina propria dendritic cells promote *de novo* generation of Foxp3 T reg cells via retinoic acid. J Exp Med. (2007) 204:1775–85. doi: 10.1084/jem.20070602, PMID: 17620362 PMC2118682

[17] Zhou T Yu Y Li L Liu X Xiang Q Yu R. Bibliometric analysis of metformin as an immunomodulator (2013-2024). Front Immunol. (2024) 15:1526481. doi: 10.3389/fimmu.2024.1526481, PMID: 39845945 PMC11750822

[18] Chen X Bahramimehr F Shahhamzehei N Fu H Lin S Wang H. Anti-aging effects of medicinal plants and their rapid screening using the nematode Caenorhabditis elegans. Phytomedicine. (2024) 129:155665. doi: 10.1016/j.phymed.2024.155665, PMID: 38768535

[19] Frasca D Diaz A Romero M Blomberg BB. Metformin enhances B cell function and antibody responses of elderly individuals with type-2 diabetes mellitus. Front Aging. (2021) 2:715981. doi: 10.3389/fragi.2021.715981, PMID: 35822013 PMC9261392

[20] Lyu Q Wen Y He B Zhang X Chen J Sun Y. The ameliorating effects of metformin on disarrangement ongoing in gastrocnemius muscle of sarcopenic and obese sarcopenic mice. Biochim Biophys Acta Mol Basis Dis. (2022) 1868:166508. doi: 10.1016/j.bbadis.2022.166508, PMID: 35905940

[21] Burdusel D Coman C Ancuta DL Hermann DM Doeppner TR Gresita A. Translatability of life-extending pharmacological treatments between different species. Aging Cell. (2024) 23:e14208. doi: 10.1111/acel.14208, PMID: 38797976 PMC11258477

[22] Hosseini Y Niknejad A Sabbagh Kashani A Gholami M Roustaie M Mohammadi M. NLRP3 inflammasomes pathway: a key target for Metformin. Inflammopharmacology. (2025) 33:1729–60. doi: 10.1007/s10787-025-01702-4, PMID: 40042723

[23] Zhang T Zhou L Makarczyk MJ Feng P Zhang J. The anti-aging mechanism of metformin: from molecular insights to clinical applications. Molecules. (2025) 30:816. doi: 10.3390/molecules30040816, PMID: 40005128 PMC11858480

[24] Wang Y Jia X Cong B. Advances in the mechanism of metformin with wide-ranging effects on regulation of the intestinal microbiota. Front Microbiol. (2024) 15:1396031. doi: 10.3389/fmicb.2024.1396031, PMID: 38855769 PMC11157079

[25] Martinez-Lopez YE Neri-Rosario D Esquivel-Hernandez DA Padron-Manrique C Vazquez-Jimenez A Sanchez-Castaneda JP. Effect of metformin and metformin/linagliptin on gut microbiota in patients with prediabetes. Sci Rep. (2024) 14:9678. doi: 10.1038/s41598-024-60081-y, PMID: 38678119 PMC11055900

[26] Jiang T Du P Liu D Chen H Ma Y Hu B. Exploring the glucose-lowering and anti-inflammatory immune mechanism of artemether by AMPK/mTOR pathway and microbiome based on multi-omics. Front Pharmacol. (2025) 16:1520439. doi: 10.3389/fphar.2025.1520439, PMID: 40046742 PMC11879814

[27] Pu D Yao Y Zhou C Liu R Wang Z Liu Y. FMT rescues mice from DSS-induced colitis in a STING-dependent manner. Gut Microbes. (2024) 16:2397879. doi: 10.1080/19490976.2024.2397879, PMID: 39324491 PMC11441074

[28] Liu Z Wu S Zhang W Cui H Zhang J Yin X. Cordycepin mitigates dextran sulfate sodium-induced colitis through improving gut microbiota composition and modulating Th1/Th2 and Th17/Treg balance. BioMed Pharmacother. (2024) 180:117394. doi: 10.1016/j.biopha.2024.117394, PMID: 39395256

[29] Livak KJ Schmittgen TD. Analysis of relative gene expression data using real-time quantitative PCR and the 2(-Delta Delta C(T)) Method. Methods. (2001) 25:402–8. doi: 10.1006/meth.2001.1262, PMID: 11846609

[30] Fontana GA Gahlon HL. Mechanisms of replication and repair in mitochondrial DNA deletion formation. Nucleic Acids Res. (2020) 48:11244–58. doi: 10.1093/nar/gkaa804, PMID: 33021629 PMC7672454

[31] Rajapakse A Suraweera A Boucher D Naqi A O’Byrne K Richard DJ. Redox regulation in the base excision repair pathway: old and new players as cancer therapeutic targets. Curr Med Chem. (2020) 27:1901–21. doi: 10.2174/0929867326666190430092732, PMID: 31258058

[32] Sui Q Yang H Hu Z Jin X Chen Z Jiang W. The research progress of metformin regulation of metabolic reprogramming in Malignant tumors. Pharm Res. (2024) 41:2143–59. doi: 10.1007/s11095-024-03783-2, PMID: 39455505

[33] Son J Cho YW Woo YJ Baek YA Kim EJ Cho Y. Metabolic reprogramming by the excessive AMPK activation exacerbates antigen-specific memory CD8(+) T cell differentiation after acute lymphocytic choriomeningitis virus infection. Immune Netw. (2019) 19:e11. doi: 10.4110/in.2019.19.e11, PMID: 31089438 PMC6494768

[34] Jafarzadeh S Nemati M Zandvakili R Jafarzadeh A. Modulation of M1 and M2 macrophage polarization by metformin: Implications for inflammatory diseases and Malignant tumors. Int Immunopharmacol. (2025) 151:114345. doi: 10.1016/j.intimp.2025.114345, PMID: 40024215

[35] Xia J Lv L Liu B Wang S Zhang S Wu Z. Akkermansia muciniphila ameliorates acetaminophen-induced liver injury by regulating gut microbial composition and metabolism. Microbiol Spectr. (2022) 10:e0159621. doi: 10.1128/spectrum.01596-21, PMID: 35107323 PMC8809353

[36] Olotu T Ferrell JM. Lactobacillus sp. for the attenuation of metabolic dysfunction-associated steatotic liver disease in mice. Microorganisms. (2024) 12:2488. doi: 10.3390/microorganisms12122488, PMID: 39770690 PMC11728176

[37] Zheng P Gao W Cong S Leng L Wang T Shi L. High-energy supplemental feeding shifts gut microbiota composition and function in red deer (Cervus elaphus). Anim (Basel). (2024) 14:1428. doi: 10.3390/ani14101428, PMID: 38791646 PMC11117297

[38] Nagano T Higashimura Y Nakano M Nishiuchi T Lelo AP. High-viscosity dietary fibers modulate gut microbiota and liver metabolism to prevent obesity in high-fat diet-fed mice. Int J Biol Macromol. (2025) 298:139962. doi: 10.1016/j.ijbiomac.2025.139962, PMID: 39826739

[39] Tilg H Moschen AR. Microbiota and diabetes: an evolving relationship. Gut. (2014) 63:1513–21. doi: 10.1136/gutjnl-2014-306928, PMID: 24833634

[40] Kim CH. Control of lymphocyte functions by gut microbiota-derived short-chain fatty acids. Cell Mol Immunol. (2021) 18:1161–71. doi: 10.1038/s41423-020-00625-0, PMID: 33850311 PMC8093302

[41] Ren H Shi Z Yang F Wang S Yuan F Li T. Deciphering unique and shared interactions between the human gut microbiota and oral antidiabetic drugs. Imeta. (2024) 3:e179. doi: 10.1002/imt2.179, PMID: 38882498 PMC11170963

[42] Forslund K Hildebrand F Nielsen T Falony G Le Chatelier E Sunagawa S. Disentangling type 2 diabetes and metformin treatment signatures in the human gut microbiota. Nature. (2015) 528:262–6. doi: 10.1038/nature15766, PMID: 26633628 PMC4681099

[43] Rosell-Diaz M Petit-Gay A Molas-Prat C Gallardo-Nuell L Ramio-Torrenta L Garre-Olmo J. Metformin-induced changes in the gut microbiome and plasma metabolome are associated with cognition in men. Metabolism. (2024) 157:155941. doi: 10.1016/j.metabol.2024.155941, PMID: 38871078

[44] Tassoulas LJ Wackett LP. Insights into the action of the pharmaceutical metformin: Targeted inhibition of the gut microbial enzyme agmatinase. iScience. (2024) 27:108900. doi: 10.1016/j.isci.2024.108900, PMID: 38318350 PMC10839685

[45] Zhao X Liu C Peng L Wang H. Metformin facilitates anti-PD-L1 efficacy through the regulation of intestinal microbiota. Genes Immun. (2024) 25:7–13. doi: 10.1038/s41435-023-00234-7, PMID: 38092885

[46] Chakraborti T Das S Mondal M Roychoudhury S Chakraborti S. Oxidant, mitochondria and calcium: an overview. Cell Signal. (1999) 11:77–85. doi: 10.1016/s0898-6568(98)00025-4, PMID: 10048784

[47] HeYShaoyongWChenYLiMGanYSunL. The functions of gut microbiota-mediated bile acid metabolism in intestinal immunity. J Adv Res. (2025). doi: 10.1016/j.jare.2025.05.015, PMID: 40354934

[48] Pi Y Mu C Gao K Liu Z Peng Y Zhu W. Increasing the hindgut carbohydrate/protein ratio by cecal infusion of corn starch or casein hydrolysate drives gut microbiota-related bile acid metabolism to stimulate colonic barrier function. mSystems. (2020) 5:e00176-20. doi: 10.1128/mSystems.00176-20, PMID: 32487741 PMC8534727

[49] Zhang Y Gao X Gao S Liu Y Wang W Feng Y. Effect of gut flora mediated-bile acid metabolism on intestinal immune microenvironment. Immunology. (2023) 170:301–18. doi: 10.1111/imm.13672, PMID: 37317655

[50] Jena PK Sheng L Di Lucente J Jin LW Maezawa I Wan YY. Dysregulated bile acid synthesis and dysbiosis are implicated in Western diet-induced systemic inflammation, microglial activation, and reduced neuroplasticity. FASEB J. (2018) 32:2866–77. doi: 10.1096/fj.201700984RR, PMID: 29401580 PMC5901391

[51] Guo C Xie S Chi Z Zhang J Liu Y Zhang L. Bile acids control inflammation and metabolic disorder through inhibition of NLRP3 inflammasome. Immunity. (2016) 45:944. doi: 10.1016/j.immuni.2016.10.009, PMID: 27760343

[52] Guan B Tong J Hao H Yang Z Chen K Xu H. Bile acid coordinates microbiota homeostasis and systemic immunometabolism in cardiometabolic diseases. Acta Pharm Sin B. (2022) 12:2129–49. doi: 10.1016/j.apsb.2021.12.011, PMID: 35646540 PMC9136572

[53] Zhao C Wu K Hao H Zhao Y Bao L Qiu M. Gut microbiota-mediated secondary bile acid alleviates Staphylococcus aureus-induced mastitis through the TGR5-cAMP-PKA-NF-kappaB/NLRP3 pathways in mice. NPJ Biofilms Microbiomes. (2023) 9:8. doi: 10.1038/s41522-023-00374-8, PMID: 36755021 PMC9908919

[54] Shi Y Su W Zhang L Shi C Zhou J Wang P. TGR5 regulates macrophage inflammation in nonalcoholic steatohepatitis by modulating NLRP3 inflammasome activation. Front Immunol. (2020) 11:609060. doi: 10.3389/fimmu.2020.609060, PMID: 33692776 PMC7937818

[55] Zhou S Hua S Chen X Ni M Liu J Wang Y. ZeXieYin formula alleviates atherosclerosis by regulating SBAs levels through the FXR/FGF15 pathway and restoring intestinal barrier integrity. Chin Med. (2025) 20:68. doi: 10.1186/s13020-025-01116-y, PMID: 40414923 PMC12103746

[56] Zhai Z Niu KM Liu H Lin C Tu Y Liu Y. Policosanol alleviates hepatic lipid accumulation by regulating bile acids metabolism in C57BL6/mice through AMPK-FXR-TGR5 cross-talk. J Food Sci. (2021) 86:5466–78. doi: 10.1111/1750-3841.15951, PMID: 34730235

[57] Cai X Cai X Xie Q Xiao X Li T Zhou T. NLRP3 inflammasome and gut microbiota-brain axis: a new perspective on white matter injury after intracerebral hemorrhage. Neural Regener Res. (2025) 21:62-80. doi: 10.4103/NRR.NRR-D-24-00917, PMID: 39885662 PMC12094575

[58] Majumder B Nataraj NB Maitreyi L Datta S. Mismatch repair-proficient tumor footprints in the sands of immune desert: mechanistic constraints and precision platforms. Front Immunol. (2024) 15:1414376. doi: 10.3389/fimmu.2024.1414376, PMID: 39100682 PMC11294168

[59] Hofer SJ Daskalaki I Bergmann M Friscic J Zimmermann A Mueller MI. Spermidine is essential for fasting-mediated autophagy and longevity. Nat Cell Biol. (2024) 26:1571–84. doi: 10.1038/s41556-024-01468-x, PMID: 39117797 PMC11392816

[60] Barcena C Valdes-Mas R Mayoral P Garabaya C Durand S Rodriguez F. Health span and lifespan extension by fecal microbiota transplantation into progeroid mice. Nat Med. (2019) 25:1234–42. doi: 10.1038/s41591-019-0504-5, PMID: 31332389

[61] Fumagalli A Castells-Nobau A Trivedi D Garre-Olmo J Puig J Ramos R. Archaea methanogens are associated with cognitive performance through the shaping of gut microbiota, butyrate and histidine metabolism. Gut Microbes. (2025) 17:2455506. doi: 10.1080/19490976.2025.2455506, PMID: 39910065 PMC11810085

[62] Gao M Li J Han X Zhang B Chen J Lang J. Effect of melatonin on gut microbiome and metabolomics in diabetic cognitive impairment. Front Pharmacol. (2024) 15:1489834. doi: 10.3389/fphar.2024.1489834, PMID: 39640487 PMC11619431

[63] Cerna C Vidal-Herrera N Silva-Olivares F Alvarez D Gonzalez-Arancibia C Hidalgo M. Fecal microbiota transplantation from young-trained donors improves cognitive function in old mice through modulation of the gut-brain axis. Aging Dis. (2025). doi: 10.14336/AD.2024.1089, PMID: 39812540 PMC12539541

[64] Pryor R Norvaisas P Marinos G Best L Thingholm LB Quintaneiro LM. Host-microbe-drug-nutrient screen identifies bacterial effectors of metformin therapy. Cell. (2019) 178:1299–312.e29. doi: 10.1016/j.cell.2019.08.003, PMID: 31474368 PMC6736778

[65] Nomura M Liu J Rovira II Gonzalez-Hurtado E Lee J Wolfgang MJ. Fatty acid oxidation in macrophage polarization. Nat Immunol. (2016) 17:216–7. doi: 10.1038/ni.3366, PMID: 26882249 PMC6033271

[66] Torres RM Turner JA D’Antonio M Pelanda R Kremer KN. Regulation of CD8 T-cell signaling, metabolism, and cytotoxic activity by extracellular lysophosphatidic acid. Immunol Rev. (2023) 317:203–22. doi: 10.1111/imr.13208, PMID: 37096808 PMC10523933

[67] Shin NR Lee JC Lee HY Kim MS Whon TW Lee MS. An increase in the Akkermansia spp. population induced by metformin treatment improves glucose homeostasis in diet-induced obese mice. Gut. (2014) 63:727–35. doi: 10.1136/gutjnl-2012-303839, PMID: 23804561

[68] Johnson SA Cambier JC. Ageing, autoimmunity and arthritis: senescence of the B cell compartment - implications for humoral immunity. Arthritis Res Ther. (2004) 6:131–9. doi: 10.1186/ar1180, PMID: 15225355 PMC464870

[69] Sato Y. Immune aging and its implication for age-related disease progression. Physiol (Bethesda). (2025) 40:0. doi: 10.1152/physiol.00051.2024, PMID: 39887318

[70] Ursini F Russo E Pellino G D’Angelo S Chiaravalloti A De Sarro G. Metformin and autoimmunity: A “New deal” of an old drug. Front Immunol. (2018) 9:1236. doi: 10.3389/fimmu.2018.01236, PMID: 29915588 PMC5994909

[71] Ramirez De Oleo I Kim V Atisha-Fregoso Y Shih AJ Lee K Diamond B. Phenotypic and functional characteristics of murine CD11c+ B cells which is suppressed by metformin. Front Immunol. (2023) 14:1241531. doi: 10.3389/fimmu.2023.1241531, PMID: 37744368 PMC10512061

[72] Liang L Yang C Liu L Mai G Li H Wu L. Commensal bacteria-derived extracellular vesicles suppress ulcerative colitis through regulating the macrophages polarization and remodeling the gut microbiota. Microb Cell Fact. (2022) 21:88. doi: 10.1186/s12934-022-01812-6, PMID: 35578339 PMC9109417

[73] Luo ZW Xia K Liu YW Liu JH Rao SS Hu XK. Extracellular Vesicles from Akkermansia muciniphila Elicit Antitumor Immunity Against Prostate Cancer via Modulation of CD8(+) T Cells and Macrophages. Int J Nanomed. (2021) 16:2949–63. doi: 10.2147/IJN.S304515, PMID: 33907401 PMC8068512

[74] Cukrowska B Motyl I Kozakova H Schwarzer M Gorecki RK Klewicka E. Probiotic Lactobacillus strains: *in vitro* and *in vivo* studies. Folia Microbiol (Praha). (2009) 54:533–7. doi: 10.1007/s12223-009-0077-7, PMID: 20140722

[75] Tyagi A Kumar V. The gut microbiota-bile acid axis: a crucial regulator of immune function and metabolic health. World J Microbiol Biotechnol. (2025) 41:215. doi: 10.1007/s11274-025-04395-7, PMID: 40555888

[76] Sun L Xie C Wang G Wu Y Wu Q Wang X. Gut microbiota and intestinal FXR mediate the clinical benefits of metformin. Nat Med. (2018) 24:1919–29. doi: 10.1038/s41591-018-0222-4, PMID: 30397356 PMC6479226

[77] Lee CB Chae SU Jo SJ Jerng UM Bae SK. The relationship between the gut microbiome and metformin as a key for treating type 2 diabetes mellitus. Int J Mol Sci. (2021) 22:3566. doi: 10.3390/ijms22073566, PMID: 33808194 PMC8037857

[78] Chang L Wang C Peng J Yuan M Zhang Z Xu Y. Sea buckthorn polysaccharides regulate bile acids synthesis and metabolism through FXR to improve Th17/Treg immune imbalance caused by high-fat diet. J Agric Food Chem. (2025) 73:15376–88. doi: 10.1021/acs.jafc.5c00409, PMID: 40470980

[79] Kiriyama Y Nochi H. The role of gut microbiota-derived lithocholic acid, deoxycholic acid and their derivatives on the function and differentiation of immune cells. Microorganisms. (2023) 11:2730. doi: 10.3390/microorganisms11112730, PMID: 38004742 PMC10672800

[80] Mohamed Elfadil O Mundi MS Abdelmagid MG Patel A Patel N Martindale R. Butyrate: more than a short chain fatty acid. Curr Nutr Rep. (2023) 12:255–62. doi: 10.1007/s13668-023-00461-4, PMID: 36763294

[81] Zhang S Sun Z Li Y Du X Qian K Yang L. Agmatine attenuates the severity of immunometabolic disorders by suppressing macrophage polarization: an *in vivo* study using an ulcerative colitis mouse model. BioMed Pharmacother. (2024) 180:117549. doi: 10.1016/j.biopha.2024.117549, PMID: 39413617

[82] Hesterberg RS Cleveland JL Epling-Burnette PK. Role of polyamines in immune cell functions. Med Sci (Basel). (2018) 6:22. doi: 10.3390/medsci6010022, PMID: 29517999 PMC5872179

[83] Wu H Esteve E Tremaroli V Khan MT Caesar R Manneras-Holm L. Metformin alters the gut microbiome of individuals with treatment-naive type 2 diabetes, contributing to the therapeutic effects of the drug. Nat Med. (2017) 23:850–8. doi: 10.1038/nm.4345, PMID: 28530702

[84] Thevaranjan N Puchta A Schulz C Naidoo A Szamosi JC Verschoor CP. Age-associated microbial dysbiosis promotes intestinal permeability, systemic inflammation, and macrophage dysfunction. Cell Host Microbe. (2017) 21:455–66.e4. doi: 10.1016/j.chom.2017.03.002, PMID: 28407483 PMC5392495

[85] Sharma R Kumar R Sharma A Goel A Padwad Y. Long-term consumption of green tea EGCG enhances murine health span by mitigating multiple aspects of cellular senescence in mitotic and post-mitotic tissues, gut dysbiosis, and immunosenescence. J Nutr Biochem. (2022) 107:109068. doi: 10.1016/j.jnutbio.2022.109068, PMID: 35618244

[86] Conway J De Jong EN White AJ Dugan B Rees NP Parnell SM. Age-related loss of intestinal barrier integrity plays an integral role in thymic involution and T cell ageing. Aging Cell. (2025) 24:e14401. doi: 10.1111/acel.14401, PMID: 39547946 PMC11896561

[87] Goyani P Christodoulou R Vassiliou E. Immunosenescence: aging and immune system decline. Vaccines (Basel). (2024) 12:1314. doi: 10.3390/vaccines12121314, PMID: 39771976 PMC11680340

[88] Ribeiro RM Perelson AS. Determining thymic output quantitatively: using models to interpret experimental T-cell receptor excision circle (TREC) data. Immunol Rev. (2007) 216:21–34. doi: 10.1111/j.1600-065X.2006.00493.x, PMID: 17367332

[89] Middelkamp V Kekalainen E. Measuring thymic output across the human lifespan: a critical challenge in laboratory medicine. Geroscience. (2025). doi: 10.1007/s11357-025-01555-3, PMID: 39946072 PMC12638580

[90] Ruddle NH Akirav EM. Secondary lymphoid organs: responding to genetic and environmental cues in ontogeny and the immune response. J Immunol. (2009) 183:2205–12. doi: 10.4049/jimmunol.0804324, PMID: 19661265 PMC2766168

[91] Boehm T Hess I Swann JB. Evolution of lymphoid tissues. Trends Immunol. (2012) 33:315–21. doi: 10.1016/j.it.2012.02.005, PMID: 22483556

[92] Soto-Heredero G Gomez de Las Heras MM Escrig-Larena JI Mittelbrunn M. Extremely differentiated T cell subsets contribute to tissue deterioration during aging. Annu Rev Immunol. (2023) 41:181–205. doi: 10.1146/annurev-immunol-101721-064501, PMID: 37126417

[93] Horna P Moscinski LC Sokol L Shao H. Naive/memory T-cell phenotypes in leukemic cutaneous T-cell lymphoma: Putative cell of origin overlaps disease classification. Cytometry B Clin Cytom. (2019) 96:234–41. doi: 10.1002/cyto.b.21738, PMID: 30328260 PMC7703846

[94] Kim YH Zhu L Pyaram K Lopez C Ohye RG Garcia JV. PLZF-expressing CD4 T cells show the characteristics of terminally differentiated effector memory CD4 T cells in humans. Eur J Immunol. (2018) 48:1255–7. doi: 10.1002/eji.201747426, PMID: 29572809 PMC6748641

[95] Attreed SE Silva C Abbott S Ramirez-Medina E Espinoza N Borca MV. A highly effective African swine fever virus vaccine elicits a memory T cell response in vaccinated swine. Pathogens. (2022) 11:1438. doi: 10.3390/pathogens11121438, PMID: 36558773 PMC9783822

[96] Zhang X Shi L Li Q Song C Han N Yan T. Caloric restriction, friend or foe: effects on metabolic status in association with the intestinal microbiome and metabolome. J Agric Food Chem. (2022) 70:14061–72. doi: 10.1021/acs.jafc.2c06162, PMID: 36263977

[97] Nair A Morsy MA Jacob S. Dose translation between laboratory animals and human in preclinical and clinical phases of drug development. Drug Dev Res. (2018) 79:373–82. doi: 10.1002/ddr.21461, PMID: 30343496

