# Supplementary figures and images for: Metformin-associated gut microbiota remodeling correlates with reinvigorated splenic immunity in aged mice: microbiome-immune crosstalk via the gut-spleen axis
Source: Front Immunol. 2025 Sep 25;16:1633486. doi: 10.3389/fimmu.2025.1633486 (PMC12507646; doi:10.3389/fimmu.2025.1633486)

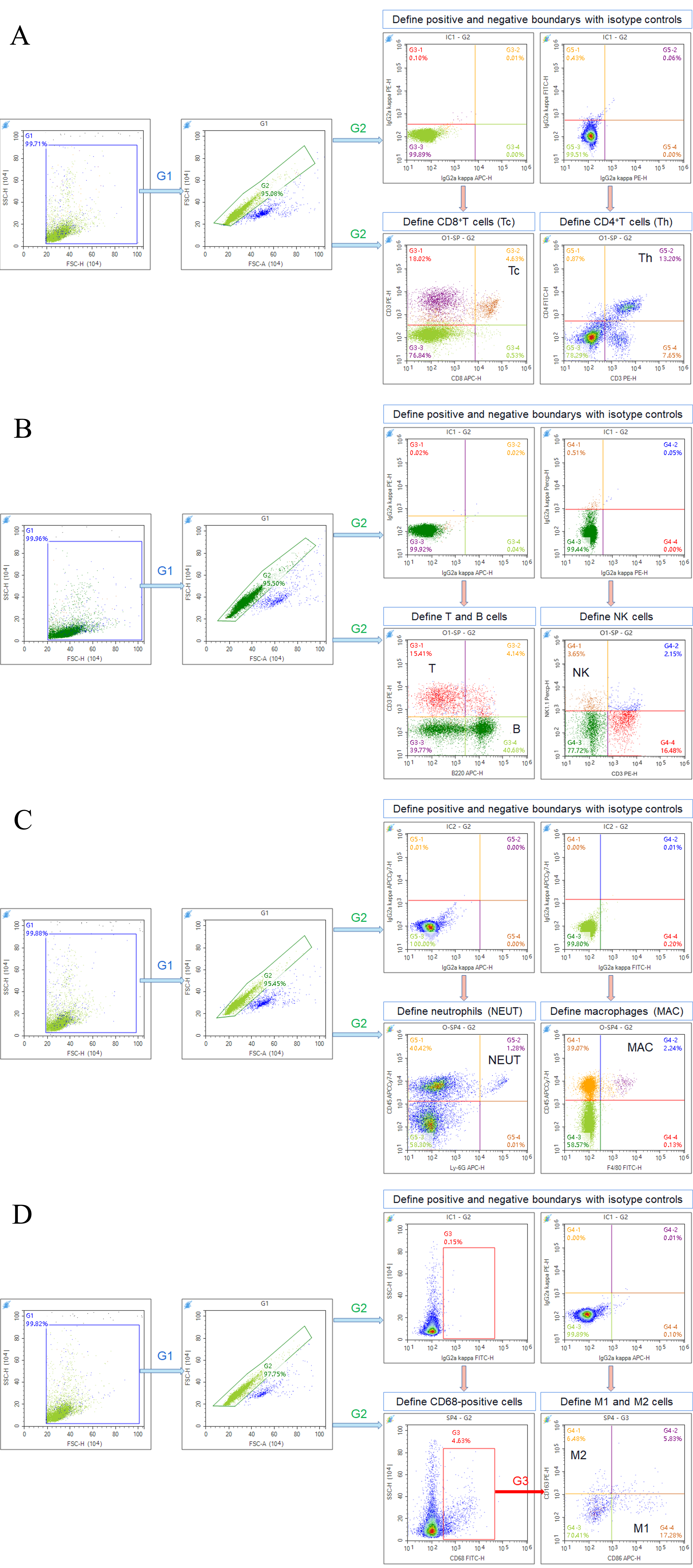

Supplement: Supplementary Figure 1 — Gating strategy for identification of immune cell populations by FCM. (A) Splenic immune cells were gated based on FSC-A/SSC-A, followed by selection of single cells using FSC-A/FSC-H. T cells were identified as CD3+, with further subdivision into CD4+ and CD8+ subsets. (B) B cells (CD3-B220+) and NK cells (CD3-NK1.1+) were identified among CD3- lymphoid cells. (C) CD45+Ly-6G+ population was defined as neutrophils and CD45+F4/80+ as macrophages. (D) CD68+ macrophages were further classified into M1 (CD68+CD86+) and M2 (CD68+CD163+) phenotypes. Fluorescence thresholds were established using isotype-matched control antibodies. [file Image1.tif]

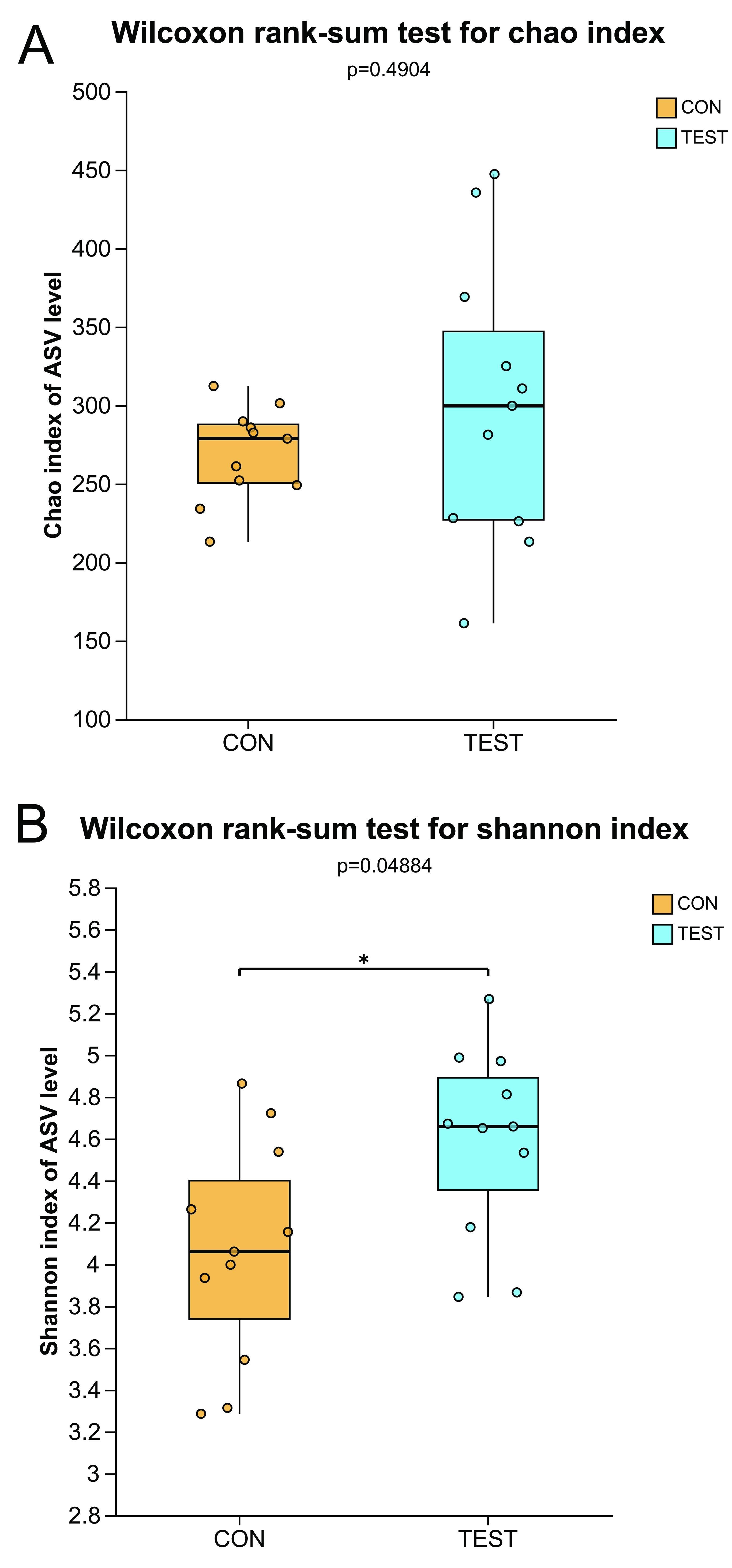

Supplement: Supplementary Figure 2 — Metformin treatment increases microbial diversity. Alpha diversity indices comparing control group (CON) and metformin-treated group (TEST). (A) Chao1 index (assessing sequencing depth), showing no significant difference (p > 0.05), Wilcoxon rank-sum test (n = 11). (B) Shannon index (assessing species diversity), showing a significant increase in the metformin group (*p<0.05), Wilcoxon rank-sum test (n = 11). [file Image2.tif]

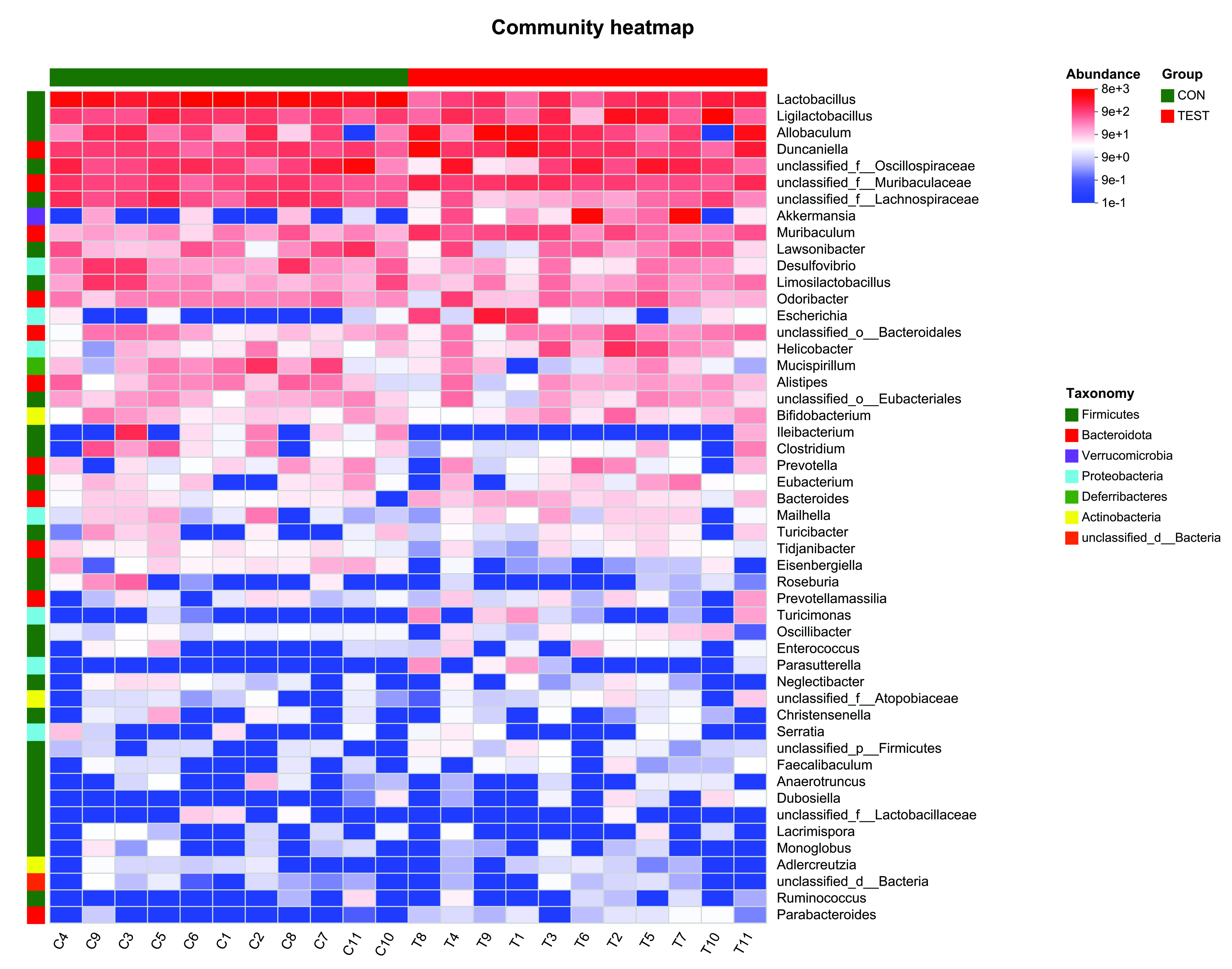

Supplement: Supplementary Figure 3 — Differential genus abundance (ANCOM-BC2) and relative abundance heatmap. CON, control group; TEST, metformin-treated group. [file Image3.tif]
